# Structural and functional rejuvenation of the aged brain by an approved anti-asthmatic drug

**DOI:** 10.1038/ncomms9466

**Published:** 2015-10-27

**Authors:** Julia Marschallinger, Iris Schäffner, Barbara Klein, Renate Gelfert, Francisco J. Rivera, Sebastian Illes, Lukas Grassner, Maximilian Janssen, Peter Rotheneichner, Claudia Schmuckermair, Roland Coras, Marta Boccazzi, Mansoor Chishty, Florian B. Lagler, Marija Renic, Hans-Christian Bauer, Nicolas Singewald, Ingmar Blümcke, Ulrich Bogdahn, Sebastien Couillard-Despres, D. Chichung Lie, Maria P. Abbracchio, Ludwig Aigner

**Affiliations:** 1 Institute of Molecular Regenerative Medicine, Paracelsus Medical University, 5020 Salzburg, Austria; 2 Spinal Cord Injury and Tissue Regeneration Center Salzburg (SCI-TReCS), Paracelsus Medical University, 5020 Salzburg, Austria; 3 Institute of Biochemistry, Emil Fischer Center, Friedrich-Alexander-University Erlangen-Nürnberg, 91054 Erlangen, Germany; 4 Center for Spinal Cord Injuries, BG Trauma Center Murnau, 82418 Murnau am Staffelsee, Germany; 5 Institute of Experimental Neuroregeneration, Paracelsus Medical University, 5020 Salzburg, Austria; 6 Department of Pharmacology and Toxicology, Institute of Pharmacy and CMBI, Leopold-Franzens-University of Innsbruck, 6020 Innsbruck, Austria; 7 Department of Neuropathology, University Hospital Erlangen, 91054 Erlangen, Germany; 8 Department of Pharmacological and Biomolecular Sciences, University of Milan, 20133 Milan, Italy; 9 Pharmidex, London W1S 1RR, UK; 10 Department for Paediatrics, Institute for Inborn Errors of Metabolism, Paracelsus Medical University, 5020 Salzburg, Austria; 11 Croatian Institute for Brain Research, University of Zagreb School of Medicine, 10000 Zagreb, Croatia; 12 Institute of Tendon and Bone Regeneration, Paracelsus Medical University, 5020 Salzburg, Austria; 13 Department of Neurology, University Hospital Regensburg, 93053 Regensburg, Germany

## Abstract

As human life expectancy has improved rapidly in industrialized societies, age-related cognitive impairment presents an increasing challenge. Targeting histopathological processes that correlate with age-related cognitive declines, such as neuroinflammation, low levels of neurogenesis, disrupted blood–brain barrier and altered neuronal activity, might lead to structural and functional rejuvenation of the aged brain. Here we show that a 6-week treatment of young (4 months) and old (20 months) rats with montelukast, a marketed anti-asthmatic drug antagonizing leukotriene receptors, reduces neuroinflammation, elevates hippocampal neurogenesis and improves learning and memory in old animals. By using gene knockdown and knockout approaches, we demonstrate that the effect is mediated through inhibition of the GPR17 receptor. This work illustrates that inhibition of leukotriene receptor signalling might represent a safe and druggable target to restore cognitive functions in old individuals and paves the way for future clinical translation of leukotriene receptor inhibition for the treatment of dementias.

## Abstract (web summary)

The leukotriene receptor antagonist montelukast is an anti-asthmatic drug. Here, the authors show that montelukast reduces neuroinflammation, promotes hippocampal neurogenesis and restores learning and memory in old rats suffering from ageing-associated cognitive dysfunction.

## Graphical abstract

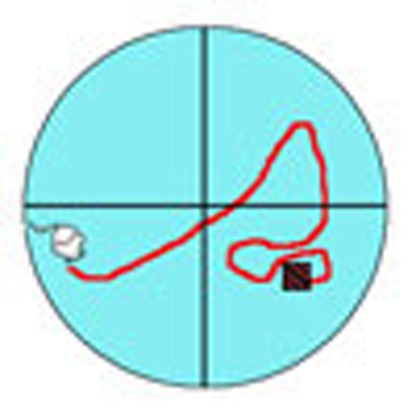

Promoting successful ageing is highly relevant, since human life expectancy has increased rapidly over the past decades. With increasing age, brain homeostasis changes, cognitive skills decline and the risk to develop dementia or neurodegenerative diseases increases dramatically^1^. Thus, a major goal of basic and applied research is to prevent or revert age-related brain changes responsible for cognitive deficits. Ultimately, this endeavour aims to maintain or restore cognitive abilities in the elderly in order to facilitate high quality of life, even in advanced age.

Histopathological hallmarks that correlate with age-related declines in cognitive function are neuroinflammation, in particular microglia dysfunction, reduced synaptic densities, blood–brain barrier (BBB) disruption and low levels of neurogenesis. Concerning neuroinflammation, microglia in the aged brain produce increased levels of pro-inflammatory cytokines such as interleukin (IL)-6, IL-1beta and tumour necrosis factor (TNF) but also of anti-inflammatory cytokines such as IL-10 and transforming growth factor (TGF) beta1 (refs 2, 3). Morphologically, aged microglia have shorter and less motile processes and larger soma sizes compared with microglia in young animals^4^. Also importantly, phagocytosis, one of the principal microglia functions, seems to be altered in the aged brain^5^, and aged microglia exhibit increased expression of the phagosomal/lysosomal associated marker CD68 (refs 6, 7). The aged brain is further characterized by a declining density of presynaptic terminals^8^, as indicated by synaptophysin immunoreactivity, for example^9^. Ample evidence of a partially leaky BBB in the aged brain is demonstrated by greater central nervous system (CNS) transfer of intravenously injected Evan's blue dye in aged compared with young adult rats^10^, and in dynamic contrast-enhanced magnetic resonance imaging studies of aged humans^11^. Moreover, the rate of adult neurogenesis, that is, the lifelong generation of new neurons from neural stem and progenitor cells in the dentate gyrus (DG) in the hippocampus and in the subventricular zone, decreases dramatically during ageing in rodents^12^, and is also reduced in elderly humans^13^.

Counteracting some, or ideally all, of such age-related changes might rejuvenate the brain and lead to preservation or even improvement of cognitive function in the elderly. The feasibility of such an approach was recently demonstrated by experiments exposing the aged brain to a young systemic environment, that is, young blood, through heterochronic parabiosis^7^,^14^. The aged brain responded to young blood by reduced microglia activation, enhanced neurogenesis, and importantly, by improved cognition. Vice versa, old blood caused premature ageing of the young brain and led to impaired cognition. A proteomic approach identified eotaxin, a chemokine involved in asthma pathology, as one of the molecules that is elevated in ageing and that contributes to neuroinflammation, reduced neurogenesis and to impaired cognition. This triggered us to hypothesize that, aside from eotaxin, additional mechanisms that are originally related to peripheral inflammatory conditions such as asthma might act or even be present in the CNS, where they potentially modulate degenerative and regenerative events.

Leukotriene signalling is well studied in the field of asthma. Leukotrienes mediate inflammatory reactions associated with increased vascular permeability^15^, and leukotriene receptor antagonists such as the drug montelukast have been successfully developed to treat asthmatic patients^16^. The role of leukotrienes in the brain, in particular their contribution to degeneration and regeneration, is less clear and sometimes even controversial^17^,^18^. Nevertheless, elevated levels of leukotrienes were reported in acute as well as chronic CNS lesions^19^,^20^, and also in the aged brain^21^, where they might mediate neuroinflammatory responses including microglia activation. Indeed, microglia express the cysteinyl leukotriene receptor CysLTR1, which mediates pro-inflammatory effects of leukotrienes, and microglia induce the expression of CysLTR2 and of the leukotriene related GPR17 receptor, for example, after ischaemia^22^,^23^. Antagonizing CysLTR1 and GPR17 with the specific inhibitor montelukast reduces the levels of inflammatory cytokine expression^22^,^23^,^24^. CysLTRs are also expressed on endothelial cells, where they mediate BBB leakage, and montelukast restores BBB integrity^25^. Furthermore, we previously demonstrated leukotriene receptor expression, in particular GPR17, in adult neurospheres, and detected a montelukast-induced dose-dependent increase in progenitor proliferation^26^. Therefore, montelukast is positioned to target at least three of the age-related cellular changes in the brain, that is, microglia activation, BBB integrity and neurogenesis.

Here, we demonstrate that montelukast reduces neuroinflammation, restores BBB integrity and increases neurogenesis specifically in the brain of old rats, the latter being mediated through inhibition of the GPR17 receptor. Most importantly, montelukast treatment restores cognitive function in the old animals, paving the way for future clinical translation for the treatment of dementias.

## Results

### Montelukast improves learning and memory in old rats

**Figure 1:**
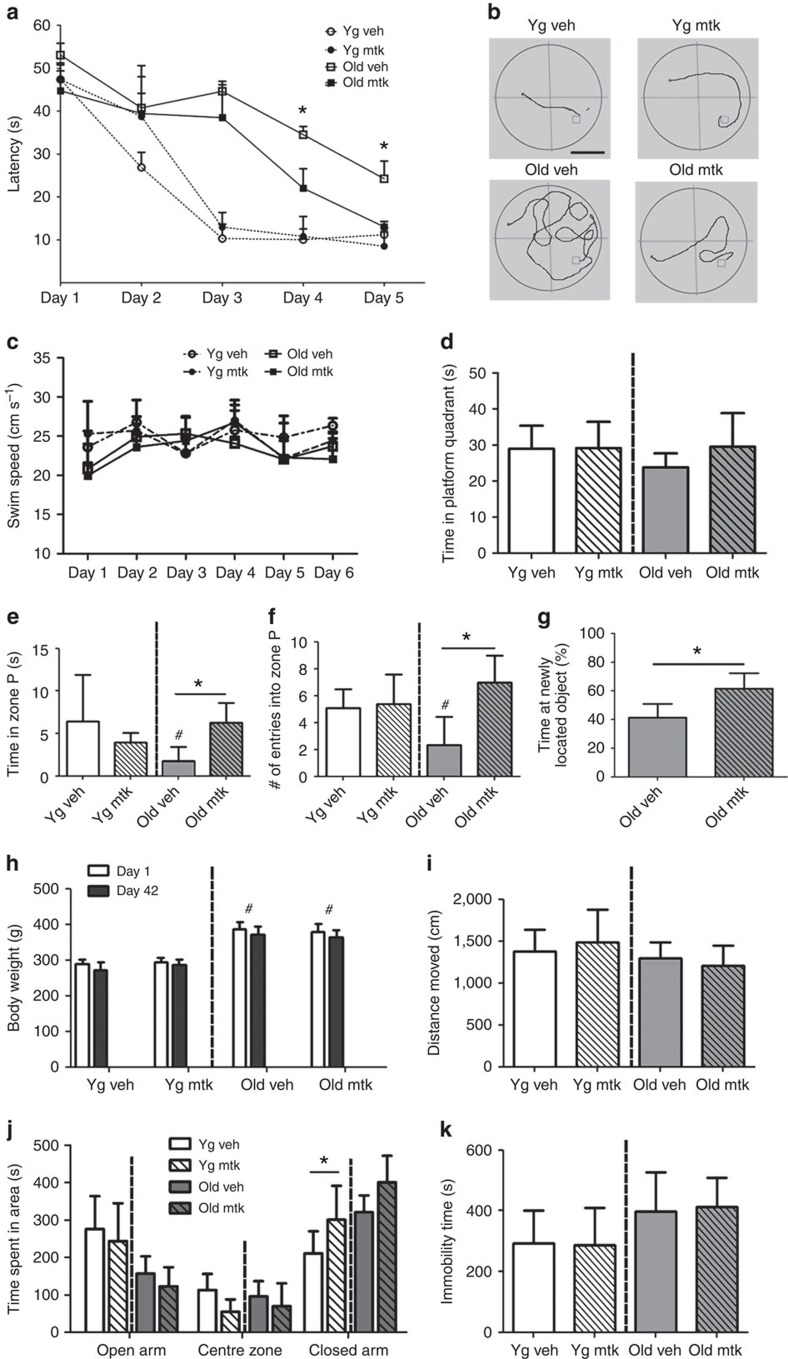
Montelukast treatment improves learning and memory in old rats. (**a**) Latency times to find the hidden platform in the Morris water maze test after daily oral administration of 10 mg kg^−1^ montelukast for 6 weeks. (**b**) Representative tracks of the distances moved in the Morris water maze on day 4 in the different age and treatment groups (scale bar, 50 cm). (**c**) Swimming speeds of animals in the Morris water maze test. (**d**–**f**) Results of probe trial on day 6: (**d**) time spent in the former platform quadrant; (**e**) time spent in zone P, a defined circular area of 20 cm diameter that encloses the former platform location; (**f**) number of entries into zone P. (**g**) Results of the object location memory test (performed on an additional cohort of rats). (**h**) Body weight of the animals on days 1 and 42 of treatment. (**i**) Locomotion as assessed by the distance moved in the open field. (**j**) Anxiety assessed in the elevated plus maze with analysis of time spent in the closed, centre and open arms. (**k**) Depression-like behaviour (immobility time in the forced swim test). Data are shown as mean±s.e.m. (**a**, **c**) or mean±s.d. (**d**–**k**). * indicates *P*<0.05; # indicates significant differences (*P*<0.05) compared to young vehicle. Two-way ANOVA (**a**,**c**,**h**,**j**), One-way ANOVA (**d**–**f**,**i**,**k**) with Bonferroni *post hoc* tests, and the unpaired Student's *t*-test (**g**) were performed. *N* per group (**a**–**f**; **h**–**k**): 10 (young vehicle), 10 (young montelukast), 7 (old vehicle), 7 (old montelukast); (**g**): 5 (old vehicle), 5 (old montelukast). yg veh, young vehicle; yg mtk, young montelukast; old veh, old vehicle; old mtk, old montelukast.

We analysed the effects of a 6-week oral montelukast (10 mg kg^−1^ body weight) treatment of young (4-months old) and old (20-months old) rats on learning and memory. Analysis of the latency times to find a hidden platform over 5 consecutive training days in a Morris water maze test, during which spatial navigation task learning is typically achieved in young animals, confirmed that old individuals have learning deficits (Fig. 1a). Montelukast treatment significantly improved task learning in old rats to a level comparable to young ones, so that on day 5 the drug-treated old animals found the platform as fast as their young counterparts. Learning in young animals was not affected by the drug treatment. Age and treatment groups only differed in the distances moved, but not in swimming speeds, excluding the latter as a possible cause of different latency times (Fig. 1b,c). Memory was assessed by removing the platform 1 day after the last learning session and by analysing the time spent in the former platform quadrant. For more precise information, we evaluated the time spent in the former platform location, and assessed the frequency entering this area. Surprisingly, old rats spent, irrespectively of the treatment, a similar amount of time in the former platform quadrant compared with their young counterparts (Fig. 1d). However, the old individuals had significant deficits in the ability to remember the exact platform location (Fig. 1e,f) suggesting specific impairments in the precise spatial memory. Montelukast treatment fully restored these memory impairments in the old rats, while it did not affect memory in the young animals (Fig. 1e,f). To explore the broader potential of montelukast in enhancing cognition in ageing, we extended our analysis and included in a different cohort of 20-months old rats a spatial discrimination task, the object location memory test. After changing the position from one of two objects whose original locations were familiar to the rats, montelukast-treated animals spent significantly more time at the newly positioned object compared with controls (Fig. 1g). Hence, these data further support that montelukast treatment leads to improved memory functions in old individuals. For drug safety reasons, and for the interest on potential effects of montelukast treatment on non-cognitive behaviours, we analysed changes in body weight (Fig. 1h), exploration and locomotion (Fig. 1i), anxiety-like behaviour (Fig. 1j) and depression-like symptoms (Fig. 1k). Montelukast treatment did not have any effect on these parameters, with the exception of a slight but significant increase in the time spent in the closed arms in the elevated plus maze in young animals (Fig. 1j), possibly pointing towards a slight anxiogenic effect of montelukast in the young group. However, the fact that the times spent in the open arms and in the centre zone in this test were similar in all groups, points away from an anxiogenic effect of montelukast.

Since it is reported that the BBB is intact in young animals but partially leaky in aged individuals, we asked if the age-dependency of the montelukast effects were due to a differential, age-related, BBB penetrance of this drug. In this context, we were also interested in the general brain penetration capacity of montelukast after oral administration, since so far, only limited BBB penetration of montelukast has been described^27^. Thus, we treated young (4 months) and old (20 months) rats for 1 week orally with montelukast (10 mg kg^−1^ body weight) and analysed its presence in serum, brain and cerebrospinal fluid (CSF) by high-performance liquid chromatography 1 h after the last montelukast administration. As expected, montelukast was present in the serum of both young and old rats. Remarkably, montelukast was, although to a lower level, also detected in the brain and in the CSF of the treated animals (Supplementary Fig. 1a). Most importantly, in a human subject taking 10 mg per day montelukast, that is, the approved dose to treat asthma, we detected montelukast in the serum and in the CSF in a similar concentration as in the rats (Supplementary Fig. 1a), suggesting that the standard 10 mg per day dose in humans is sufficient to reach a therapeutic dose in the CSF. In addition, a re-analysis of the original CNS pharmacology data of montelukast^27^ indicates a significant BBB penetrance of the drug (Supplementary Fig. 1b). These data clearly demonstrate that orally administered montelukast does cross the BBB in a therapeutic dose, and that age-dependent differential BBB integrity does not affect the capacity of montelukast to enter the brain.

### Montelukast reduces microglia reactivity in old rats

**Figure 2:**
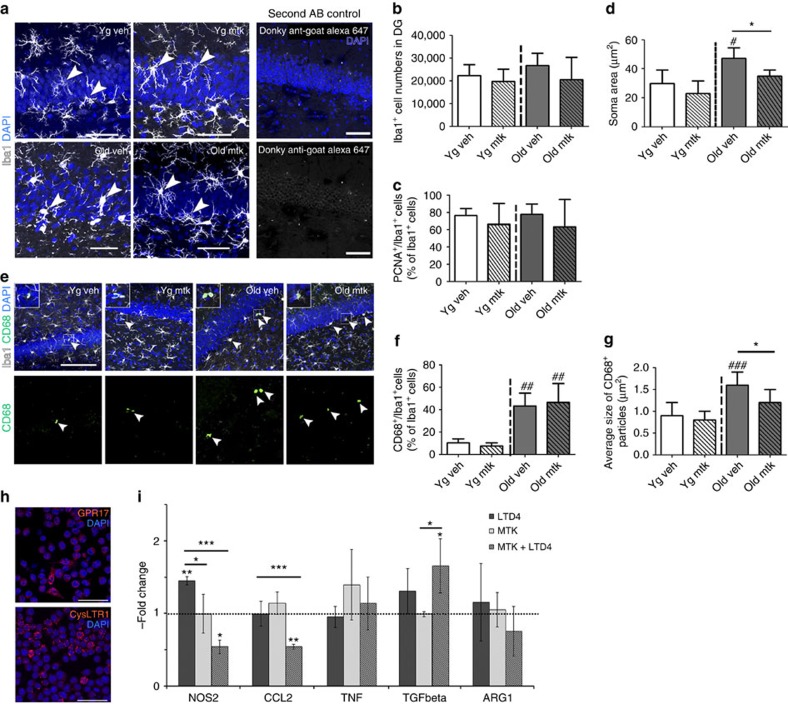
Montelukast modulates microglia in old rats and neuroinflammatory gene expression. (**a**) Iba1^+^ immunostaining in the DG of the different age- and treatment groups (white arrowheads point toward representative Iba1^+^ cells). Control stainings, in which the primary goat Iba1 antibody was omitted, excluded unspecific labelling of the secondary antibody. (**b**) Total numbers of Iba1^+^ cells, and (**c**) percentages of proliferating microglia (Iba1^+^/PCNA^+^) in the DG of young (4 months) and old (20 months) rats after a 6-week treatment with montelukast (10 mg kg^−1^ daily p.o.). (**d**) Soma sizes of Iba1^+^ cells in the DG of young and old montelukast- and vehicle-treated rats. (**e**) Representative images of CD68^+^ immunostaining in Iba1^+^ microglia (arrowheads) of the different experimental groups. (**f**) Percentage of Iba^+^ cells that contain CD68^+^ particles. (**g**) Average size of CD68^+^ particles in Iba1^+^ cells of in the DG. (**h**) Immunoreactivity for CysLTR1 and for GPR17 in BV-2 cells. (**i**) mRNA expression of NOS2, CCL2, TNF, TGF-beta1 and of ARG1 in BV-2 cells 24 h after treatment with 100 nM LTD4 and/or with 15 μM montelukast, and with vehicle control. N per group in (**b**–**d**,**f**,**g**): 10 (young vehicle), 10 (young montelukast), 7 (old vehicle), 7 (old montelukast). Every tenth section (400 μm interval) of one brain hemisphere (**b**,**c**) or four sections (**d**,**f**,**g**) per animal were analysed. In (**i**), 3–5 biological replicates were performed in technical duplicates. Data are shown as mean±s.d. *P<0.05, **P<0.01, ***P<0.001. Significant differences between the old age groups and the young vehicle group were indicated by # (#=*P*<0.05; ###=*P*<0.001). One-way ANOVA (**b**–**d**,**f**,**g**) and Two-way ANOVA (**i**) with Bonferroni *post hoc* tests were performed. Scale bars, 50 μm (**a**); 100 μm (**e**). yg veh, young vehicle; yg mtk, young montelukast; old veh, old vehicle; old mtk, old montelukast; DG, dentate gyrus.

As spatial learning and memory are strongly associated with hippocampal function, we concentrated our further histological analyses on this region. As a measure of neuroinflammation, we first analysed the number of Iba1^+^ microglia, their proliferation index (% of proliferating cell nuclear antigen (PCNA)^+^ cells) and their soma size, the latter being repeatedly used as a surrogate marker for microglia activation, in particular of phagocytic activity^28^. The number of Iba1^+^ cells and their proliferation index was similar in all groups (Fig. 2b,c). However, compared with the young age groups, microglia somata were significantly larger in old rats treated with vehicle only. Importantly, the 6-week montelukast treatment completely reverted this age-related phenotype, but did not affect microglia in the young rats (Fig. 2a,d). Next, we speculated whether the increased microglia soma size in old rats, as well as its reduction by montelukast, are linked to changes in phagocytosis. We therefore analysed the expression pattern of CD68, a protein associated to the phagosomal/lysosomal pathway of microglia^29^. Old animals had a significant higher number of Iba1^+^ microglia co-expressing CD68 (Fig. 2f) and bigger CD68^+^ particles (Fig. 2e,g) compared with the young animals, suggesting altered phagosomal/lysosomal processing of microglia in old rats. Although montelukast did not influence the number of Iba1^+^/CD68^+^ cells in the old age-group, drug treatment led to a significant reduction of the average CD68 particle size in the old animals. In young animals, neither the number of CD68^+^ expressing microglia, nor the average particle size were affected by montelukast.

Addressing more specifically the pattern of neuroinflammatory gene expression, we made use of the BV-2 microglia cell line. First, BV-2 cells showed immunoreactivity for the target receptors of montelukast, the leukotriene receptor CysLTR1 and the GPR17 receptor (Fig. 2h). Second, we treated BV-2 cells for 24 h with 100 nM of the receptor ligand leukotriene D4 (LTD4), with 15 μM montelukast, or with 100 nM LTD4 together with 15 μM montelukast, and analysed mRNA expression of inflammatory and anti-inflammatory genes (Fig. 2i). A 24-h treatment with LTD4 led to a significant upregulation of the expression of the pro-inflammatory enzyme NOS2. 24 h co-stimulation with montelukast counteracted this effect and significantly reduced NOS2 mRNA expression. Moreover, in the presence of LTD4 montelukast downregulated the expression of the monocyte-recruiting cytokine CCL2, whose expression is elevated in ageing^7^. Also, mRNA expression of the neuroprotective cytokine TGF-beta1 was significantly elevated after 24 h treatment with montelukast in the presence of LTD4. In summary, these findings further add to the established mode of action of montelukast as an anti-inflammatory drug.

Next, as the BBB is disrupted in neuroinflammation and in ageing^30^, we analysed BBB integrity using the tight junction protein claudin-5. Endothelial cells in the hippocampus of young (4 months) vehicle-treated rats showed strong and continuous immunostaining with an anti-claudin-5 antibody, illustrating an intact BBB (Supplementary Fig. 2). In contrast, claudin-5 expression in endothelial cells of old (20 months) vehicle-treated rats was diffuse and weak, which points toward a disrupted BBB. The old montelukast-treated rats showed more intense, more continuous and better-defined endothelial claudin-5 staining compared with age-matched vehicle controls, indicating that montelukast improved BBB integrity in old rats.

### Montelukast restores hippocampal neurogenesis in old rats

**Figure 3:**
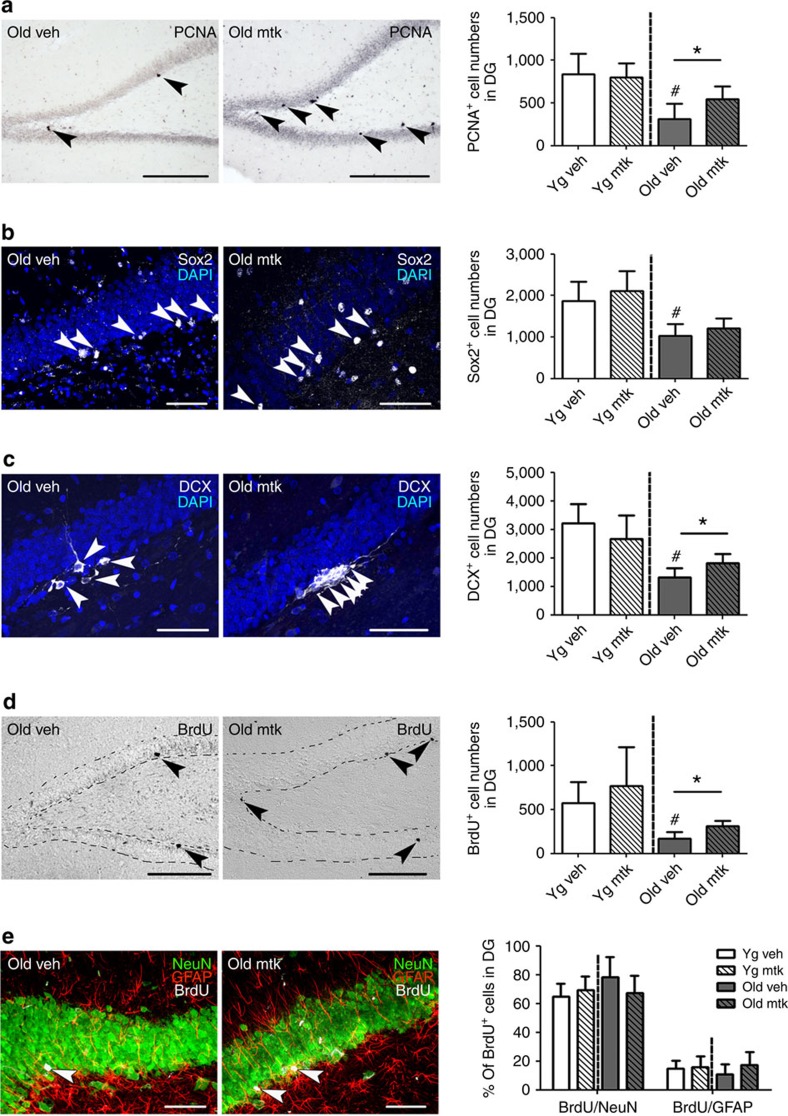
Montelukast treatment increases dentate gyrus neurogenesis in old rats. (**a**) Number of PCNA^+^ cell numbers in the DG. (**b**) Number of Sox2^+^ neural stem cells in the subgranular zone of the DG. (**c**) DCX^+^ cell numbers in the DG. (**d**) Number of BrdU^+^ cells 4 weeks after BrdU labelling of the cells. (**e**) The percentages of BrdU^+^ cells that have differentiated into neurons (BrdU^+^/NeuN^+^) or astrocytes (BrdU^+^/GFAP^+^). Arrowheads point to cells stained positively for the respective marker. Pictures illustrate representative images of antibody labelling in the DG of old vehicle and old montelukast-treated rats. Scale bars, 200 μm (**a**, **d**), 100 μm (**b**, **c**, **e**). Data are shown as mean±s.d. * indicates *P*<0.05; # indicates significant differences (*P*<0.05) specifically compared with young vehicle. One-way ANOVA with Bonferroni *post hoc* tests was performed. *N* per group: 10 (young vehicle), 10 (young montelukast), 7 (old vehicle), 7 (old montelukast). Every tenth section (400 μm interval) of one brain hemisphere per animal was analysed. yg veh, young vehicle; yg mtk, young montelukast; old veh, old vehicle; old mtk, old montelukast; DG dentate gyrus.

Further, we investigated if the 6-week treatment with montelukast had any effects on DG neurogenesis. Proliferation (number of PCNA^+^ cells in the subgranular and granular zone) was approximately three-times lower in old compared with young animals. Montelukast treatment significantly enhanced proliferation, and this effect was specific for the old individuals (Fig. 3a). The surplus in PCNA^+^ cells in the old montelukast-treated animals was apparently not due to an enlargement of the neural stem cell pool, as the number of subgranular Sox2^+^ cells was unchanged by the drug treatment (Fig. 3b). By contrast, it was the pool of DCX^+^ young immature neurons that was significantly expanded by the montelukast treatment (Fig. 3c). The additional newly generated cells in the old montelukast-treated rats survived for at least 4 weeks as indicated by the number of cells that had incorporated BrdU two weeks after onset of treatment (Fig. 3d). The effects of montelukast on proliferation and survival resulted in a significant increase of BrdU^+^ newly generated cells in the DG of old animals after the 6-week treatment. The fate of the newly generated cells (percentage of BrdU^+^ cells co-labelling with NeuN for mature neurons or with GFAP for astrocytes) was in the expected range of 60–80% neuronal and ∼15% glial differentiation and was neither affected by age nor by treatment (Fig. 2e). As a net result, montelukast treatment significantly increased the number of newly generated neurons in the DG of old rats (215.97±60.04 BrdU^+^/NeuN^+^ cells) compared with age-matched controls (131.48±44.16 BrdU^+^/NeuN^+^ cells).

Since ageing is associated with loss of presynaptic terminals^8^,^9^ and altered neuronal functionality^31^, we explored the possibility that the montelukast-induced effect on cognitive function was related to these parameters. Indeed, synaptic density (synaptophysin positive area) was significantly reduced in the hippocampus of old rats, but only slightly and not significantly in the cortex. Montelukast treatment did not restore the synaptic density in the hippocampus (Supplementary Fig. 3a) implying that the cognitive improvements in old rats were not related to changes in synaptic densities in the DG. In the same context, the 6-week montelukast treatment did neither change the DG immunohistochemical expression pattern of c-Fos, an immediate early gene and marker for activated neurons (Supplementary Fig. 3b), nor did montelukast and/or leukotrienes alter neuronal network activity in rat hippocampal cultures (Supplementary Fig. 3c–g).

**Figure 4:**
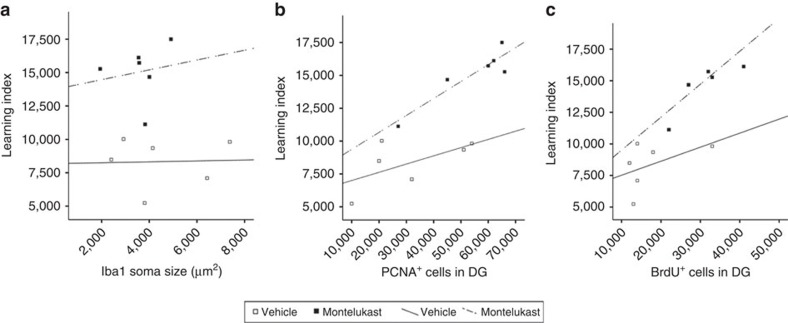
Montelukast-mediated cognitive improvements in old rats correlate with increased neurogenesis. (**a**) Correlation analysis showed that the individual learning scores of old vehicle-treated and old montelukast-treated rats were independent of the soma size of Iba1^+^ cells in the DG (vehicle: *R*^2^=0.001; *y*=81.79+0.034*x; montelukast: *R*^2^=0.027; *y*=137.35 +0.368*x). (**b**) The correlation coefficient between learning success and number of PCNA^+^ cells revealed a tendency towards correlation in the old vehicle-treated rats (R^2^=0.361; y=63.75+0.063*x). Montelukast provoked a stronger correlation and led to a steeper slope of the regression line (*R*^2^=0.843; *y*=80.8+0.129*x). (**c**) Similarly, learning and the number of BrdU^+^ cells showed a trend for correlation in vehicle-treated old rats (*R*^2^=0.225; *y*=64.11+0.111*x), and again, this correlation was strengthened in montelukast-treated rats (*R*^2^=0.601; *y*=69.01+0.261*x). N per group: 6 (old vehicle), 6 (old montelukast). Correlation analysis was performed using the Pearson product moment correlation test. DG, dentate gyrus.

Taken together, montelukast treatment reduced microglia activation, elevated neurogenesis, and restored cognitive function in old rats. Regression and correlation analyses showed that learning did not correlate with microglial soma size (*R*^2^=0.001) and that the montelukast-induced learning improvement was independent of the changes in microglia morphology in the old animals (*R*^2^=0.027) (Fig. 4a). In contrast, correlation coefficients of *R*^2^=0.306 for learning/number of PCNA^+^ cells in the DG, and of R^2^=0.225 for learning/number of BrdU^+^ cells in old vehicle-treated rats, let rather suggest that the learning success of old rats might depend on the rate of neurogenesis (Fig. 4b,c). Moreover, montelukast treatment provoked a stronger correlation between learning and cell proliferation (R^2^=0.843) and led to a steeper slope of the regression line, and also strengthened the correlation between learning/number of BrdU^+^ cells (R^2^=0.601). This might allow the hypothesis that montelukast not only increases the generation of new neurons but also might improve the functional use of these neurons.

### Leukotriene signalling in the ageing brain

**Figure 5:**
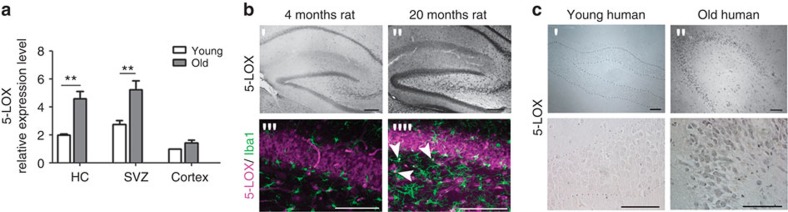
5-LOX expression is upregulated in the hippocampus of old rats and elderly humans. (**a**) 5-LOX mRNA expression was significantly elevated within the neurogenic regions (hippocampus: 2.4-fold; SVZ: 1.8-fold) of old (20 months) compared to young (4 months) rats (*n*=3 per group). (**b**) 5-LOX immunoreactivity was strongly increased in the DG of old ('') compared with young (') rats. Although in young rats 5-LOX staining was predominately allocated to granular neurons ('''), in old rats also Iba1^+^ microglia ('''', arrowheads) expressed 5-LOX. Pictures illustrate representative 5-LOX stainings; five rats per group were analysed. (**c**) In elderly humans (>60 years) (''), intensity of 5-LOX immunostaining in the DG was clearly upregulated compared with young persons (<35 years) (') (lower panels show higher magnifications of the dentate gyri depicted in c',''). Images in c illustrate the DG of a 27 year old human person ('), representing the young age group (hippocampi from five persons<35 years analysed), and the DG of a 67-year-old person (''), representing 5-LOX expression in the elderly group (hippocampi from five persons >60 years analysed). Data are shown as mean±s.d.; ** indicates *P*<0.01; Student's unpaired *t*-test (**a**). Scale bars, 100 μm (**b**,**c**). DG, dentate gyrus; HC hippocampus; SVZ, subventricular zone.

Since montelukast was developed as a leukotriene receptor inhibitor, we hypothesised that leukotriene production might correlate with the herein described age-associated brain changes. First, we analysed the mRNA expression of 5-LOX, the key enzyme in leukotriene synthesis, in different brain regions of young and old rats. We found increased levels of 5-LOX mRNA in the neurogenic regions (hippocampus and subventricular zone), but not in the cortex, of old rats (Fig. 5a). Age-related elevation of 5-LOX expression was also evident at the protein level as illustrated by immunohistochemistry. 5-LOX immunoreactivity was more intense in the hippocampus of old compared with young rats (Fig. 5b) and was localised to DG granular neurons and to some microglia (Fig. 5b). Importantly, 5-LOX immunoreactivity was also strongly enhanced in the DG of elderly humans (>60 years) (Fig. 5c'') compared with young ones (<35 years) (Fig. 5c').

**Figure 6:**
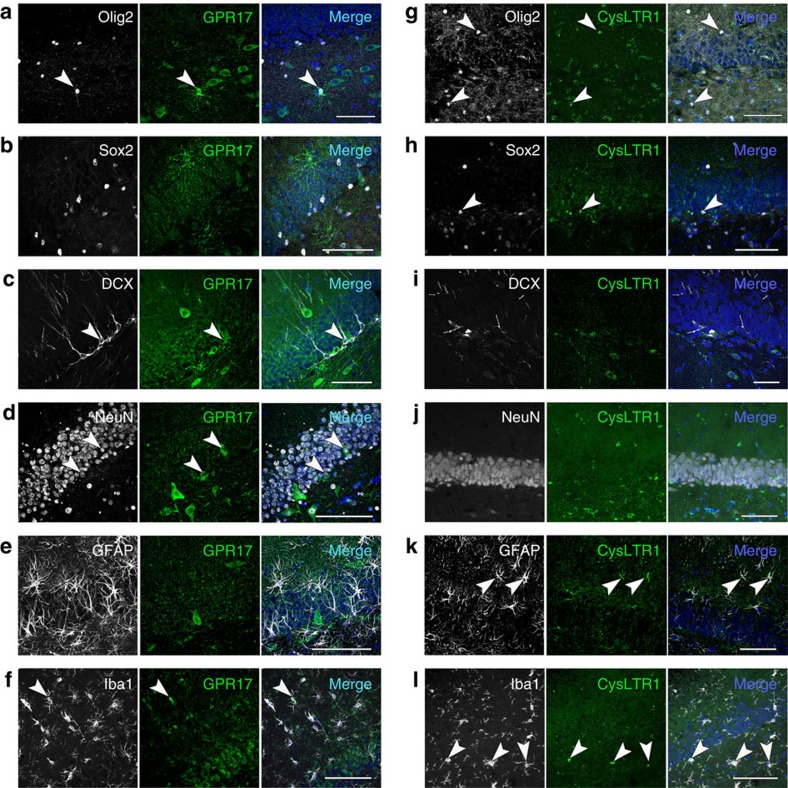
GPR17 and CysLTR1, target receptors of montelukast, are expressed within the dentate gyrus of old rats. (**a**–**f**) GPR17 expression pattern in the DG of old (20 months) rats. GPR17 immunoreactivity was localised to cells of the oligodendroglial lineage (Olig2^+^), **a**), to some DCX^+^ neuronal progenitors (**c**), to a subset of NeuN^+^ granular neurons (**d**) and to a fraction of Iba1^+^ microglia (**f**). GPR17 expression was neither observed in Sox2^+^ neural stem cells (**b**) nor in GFAP^+^ astrocytes (**e**). (**g**–**l**) CysLTR1 immunoreactivity in the DG of 20-month old rats was localised to a few Sox2^+^ cells (**h**), to cells of the oligodendroglial lineage (Olig2^+^, **g**), to GFAP^+^ astrocytes (**k**), and to some Iba1^+^ microglia (**l**). CysLTR1 expression was not observed in DCX^+^ neuronal progenitors (**i**) or in NeuN^+^ granular neurons (**j**). Scale bars, 50 μm (**a**–**l**). Arrowheads indicate receptor co-localisation. DG, dentate gyrus.

Regarding the targets of montelukast, we and others recently demonstrated CysLTR and GPR17 mRNA expression in adult neurosphere cultures^26^,^32^. Here, we analysed immunoreactivity of GPR17 and CysLTR1 in the hippocampal DG of old rats (Fig. 6) and in adult hippocampal neurosphere-derived cells (Supplementary Fig. 4). GPR17 was localised to progenitors and mature cells of the oligodendroglial (Olig2, RIP) and neuronal (DCX, NeuN, betaIII-tubulin) lineage, as well as to some microglia cells (Iba1). We excluded GPR17 expression in neural stem cells (subgranular Sox2) and in astrocytes (GFAP). In contrast, CysLTR1 expression was predominantly observed on astrocytes (GFAP), on microglia (Iba1) and on some oligodendroglial cells (Olig2, RIP). CysLTR1 was only rarely detected on neural stem cells (Nestin, Sox2) and was absent on cells of the neuronal lineage (DCX, NeuN, betaIII-tubulin). Hence, both *in vitro* and *in vivo*, CysLTR1 expression was primarily found on non-neuronal cells, while GPR17 was predominantly present on cells of the neuronal lineage, suggesting that the montelukast-induced effects on neurogenesis might most likely be mediated through inhibition of GPR17 and not CysLTR1.

**Figure 7:**
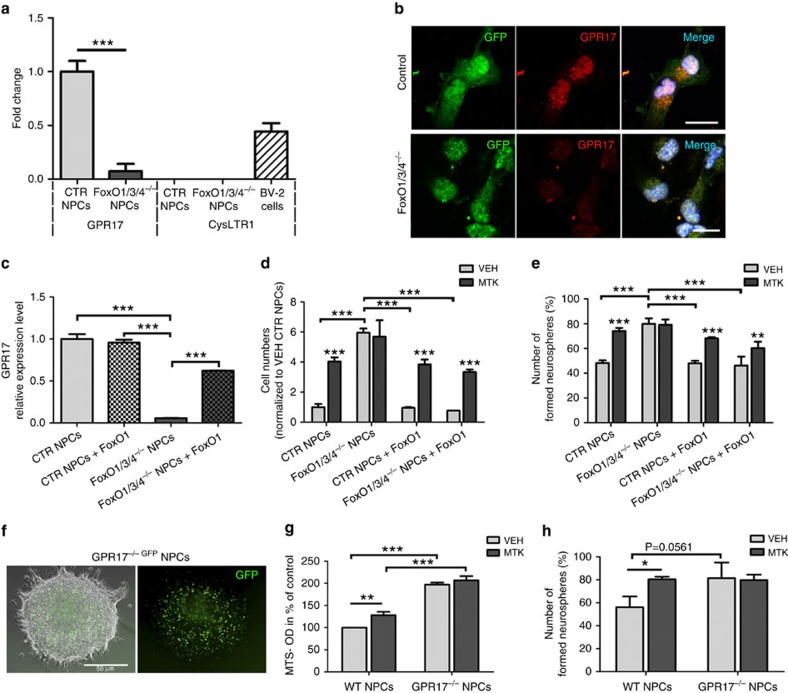
GPR17 knockdown and knockout in neurospheres induce hyperproliferation and abolish the effects of montelukast. (**a**) GPR17 mRNA expression in mouse neurospheres derived from adult *FoxO1,3,4*^*fl/fl*^ mice and recombined with GPF/Cre retrovirus (*FoxO1/3/4*^*−/−*^ *NPCs*), compared with neurospheres infected with GFP-only retrovirus (*control; CTR NPCs*). CysLTR1 mRNA was not expressed in mouse neurospheres and not affected by the FoxO1,3,4 knockout. BV-2 cells served as positive control for CysLTR1 primer specificity. (**b**) GPR17 immunoreactivity in GFP^+^ cells of *FoxO1/3/4*^*−/−*^ neurospheres compared with control neurospheres. (**c**) Overexpression of FoxO1 by lentiviral transfection upregulated GPR17 mRNA expression in *FoxO1,3,4*^*−/−*^ neurospheres (*FoxO1/3/4*^*−/−*^ *NPCs +FoxO1*) to 60%. (**d**) Proliferative activity of FoxO1,3,4^−/−^ neurospheres assessed by a neurosphere bulk assay. Cell numbers were 6 × higher in FoxO1/3/4^−/−^ neurospheres compared with controls. A 7-day montelukast treatment (10 μM) significantly increased cell numbers in control neurospheres, but did not affect FoxO1/3/4^−/−^ neurospheres. FoxO1 overexpression significantly reduced cell numbers in FoxO1,3,4^−/−^ neurospheres; Montelukast treatment provoked a significant elevation of cell numbers in the FoxO1 overexpressed FoxO1,3,4^−/−^ neurospheres. (**e**) In a single-cell neurosphere assay, neural stem cells with FoxO1/3/4 deletion generated significantly more neurospheres than control cells. A 7-day montelukast treatment (10 μM) significantly elevated the number of neurospheres in control, but not in *FoxO1/3/4*^*−/−*^ cells. FoxO1 overexpression decreased neurosphere numbers in vehicle-treated *FoxO1/3/4*^*−/−*^ NPCs. Montelukast treatment increased the numbers of neurospheres in FoxO1 transfected *FoxO1/3/4*^*−/−*^ NPCs compared with vehicle. (**f**) In neurospheres isolated from adult *GPR17*^*−/− GFP*^ mice, high numbers of GFP^+^ cells are present, indicating active transcription of the *GPR17* gene locus in these cells. (**g**) Optical density (OD) in an MTS assay was 97% increased in *GPR17*^*−/−*^ neurospheres compared with wild-type (WT) neurospheres. A 7-day treatment with 10 μM montelukast significantly elevated OD absorbance in wild-type NPCs, but did not affect *GPR17*^*−/−*^ cells. (**h**) In the single-cell neurosphere assay, neural stem cells from *GPR17*^*−/−*^ neurospheres generated more neurospheres than control cells (*P*=0.0561). Montelukast (10 μM, 7 days) significantly elevated neurosphere numbers in control, but not in *GPR17*^*−/−*^ NPCs. Three independent experiments were done in triplicates. Data are shown as mean±s.d. (**a**,**c**–**e**,**g**,**h**). * indicates *P*<0.05, ** indicates *P*<0.01. Student's unpaired *t*-test (**a**) and Two-way ANOVA (**c**–**e**,**g**,**h**) with Bonferroni *post hoc* tests were performed. Scale bars (**b**,**f**), 50 μm. MTK, montelukast; NPC, neuronal progenitor cells; VEH, Vehicle.

Since (i) the montelukast-induced improvement in cognition correlated best with the elevation of neurogenesis, and (ii) neuronal progenitors and differentiated neurons were devoid of CysLTR1 but expressed GPR17, we focused the further analysis on GPR17. It is reported that GPR17 is a target of FoxO transcription factors, and that GPR17 expression requires FoxO1^33^. Here, we first used *FoxO1,3,4*^*fl/fl*^ mouse-derived neurospheres that were recombined with GFP/Cre retrovirus and FACS sorted for GFP^+^ cells to get first insight into a potential role of GPR17 in neurogenesis. We could demonstrate a drastic reduction of GPR17 mRNA and protein intensity in the recombined cells compared with control–infected cells (Fig. 7a,b). Further, in line with our present immunohistochemical observations and our previously published findings^26^, neither control nor *FoxO1,3,4*^*−/−*^ neurospheres expressed CysLTR1 mRNA (Fig. 7a). Importantly, overexpression of FoxO1 by lentiviral transfection was sufficient to rescue GPR17 expression and increased its mRNA expression in FoxO1,3,4 deleted neurospheres to 62% of control levels. In contrast, control neurospheres did not further upregulate GPR17 mRNA in response to FoxO1 overexpression (Fig. 7c).

Addressing neuronal progenitor cell (NPC) proliferation in a neurosphere bulk assay, we observed a six times elevated expansion rate in *FoxO1,3,4*^*−/−*^ neurospheres compared with control neurospheres, which in contrast to the control NPCs, was not further potentiated by montelukast (Fig. 7d). Overexpression of FoxO1 in the FoxO1,3,4 deleted cells (which, as described above, led to upregulation of GPR17 mRNA, Fig. 7c) significantly reduced the expansion rate to a level that was comparable to control NPCs. Interestingly, FoxO1 transfected *FoxO1,3,4*^*−/−*^ neurospheres were susceptible to montelukast treatment and showed a significant increase of cell numbers compared to vehicle-treatment. In clonal analyses, the percentage of neurosphere-forming cells was significantly elevated after FoxO1,3,4 deletion. Moreover, while montelukast treatment elevated the clonal growth rate of control cells, it did not further affect the growth of FoxO1,3,4 deleted cells (Fig. 7e). FoxO1 overexpression in vehicle-treated *FoxO1,3,4*^*−/−*^ NPCs decreased neurosphere numbers, and again, these cells reacquired the ability to respond to montelukast by increased numbers of neurospheres, compared with vehicle.

Next, to analyse more specifically the role of GPR17 in neural progenitor proliferation *in vitro*, we used neurospheres isolated from adult *GPR17*^*−/− GFP*^ mice. GFP expression in a high number of neurosphere cells indicated transcriptional activity at the *GPR17* gene locus in NPCs (Fig. 7f). We observed a 96% increased basal proliferative activity in an MTS assay in *GPR17*^*−/−*^ neurosphere cultures compared with wild-type neurospheres, and interestingly, in contrast to wild-type cells, these *GPR17*^*−/−*^ cells did not respond to montelukast with enhanced proliferation (Fig. 7g). Similarly, in a single-cell neurosphere assay, cells from dissociated *GPR17*^*−/−*^ neurospheres generated more neurospheres than wild-type cells (*P*=0.0561; Two-way analysis of variance (ANOVA), Bonferroni *post hoc* test), and the increase in neurospheres number induced by montelukast in wild-type cells was absent in knockout cells (Fig. 7h). In conclusion, neurospheres with a genetically induced constraint in GPR17 expression, mediated either through a FoxO1,3,4 deletion or a direct GPR17 knockout, are released from growth restriction and do not further respond to montelukast with enhanced proliferation, suggesting that the montelukast-induced effect on neurogenesis might indeed be mediated through inhibition of GPR17.

## Discussion

The present work demonstrates that the anti-asthmatic drug and leukotriene receptor antagonist montelukast restores learning and memory function in old rats, in which cognition is compromised as a result of the physiological ageing process. Other general activities and psychiatric behaviours were not affected by the drug treatment in the old animals.

Remarkably, montelukast serum levels of the rats used in this study (young: 288±57.18 ng ml^−1^; old 365±56.45 ng ml^−1^; Supplementary Fig. 1) were almost identical to the maximum plasma concentrations in humans after oral administration of the clinical dose of 10 mg montelukast daily (385±85 ng ml^−1^)^27^, illustrating that the animals were treated with montelukast in a dose that pharmacologically resembles the one that is approved for its use in humans. A critical issue for montelukast to be developed as a CNS drug is, of course, its CNS pharmacokinetics. Although montelukast was so far always considered as a drug with only limited CNS penetration, careful re-analysis of the original pharmacokinetic report on montelukast^27^ reveals that one hour after i.v. drug administration, a substantial amount of radioactive equivalents of [C14] montelukast (∼1/10 of the plasma levels) had reached the brain (Supplementary Fig. 1b). Most remarkably, while in plasma (and most other organs, for example, lung and muscle) montelukast levels strongly decreased within 24 h, the amount of montelukast in the brain increased. As a consequence, 24 h after drug injection, montelukast levels in the brain were even higher than in plasma (Supplementary Fig. 1b), suggesting the existence of an active transport mechanism for montelukast through the BBB. Indeed, montelukast is taken up from the intestine into the blood stream by the organic anion-transporting polypeptide (OATP)2B1, a transporter that is expressed also by endothelial cells of brain capillaries^34^. Also, the majority (99%) of montelukast in plasma is bound to proteins, mainly albumin^27^, providing a BBB transport mechanism as albumin has been shown to act as a carrier through the BBB^35^. The potential of montelukast to enter the CNS is further strongly supported by our present pharmacokinetic results obtained from rats (Supplementary Fig. 1a). Strikingly, montelukast was also detected in the CSF in a human asthma patient, who was on the approved 10 mg per day dose of montelukast, and levels in serum and CSF were almost identical to the concentrations found in rats treated with 10 mg kg^−1^ montelukast (Supplementary Fig. 1a). Entry of montelukast into the CNS is further supported by the plethora of preclinical data on the effects of systemic montelukast treatment on brain structure and function. In various animal models of neurodegenerative diseases, including a model of kainic acid-induced loss of memory function, an acute Huntintgon's disease model of quinolinic acid and malonic acid injection-induced degeneration of striatal neurons, and a β-amyloid injection model of Alzheimer's disease, treatment with montelukast attenuated behavioural deficits^36^,^37^,^38^, which was accompanied by structural brain changes such as inhibition of neuroinflammation and reduced neuronal cell death^37^,^38^. Neuroprotective effects of montelukast have also been demonstrated in animal models of acute CNS injuries and stroke. For example, in the middle cerebral artery occlusion model of stroke, pre-treatment with montelukast significantly attenuated neurological deficits, infarct volumes, brain oedema, loss of neurons and BBB disintegrity^39^. In a rat model of spinal cord injury, montelukast treatment resulted in neuroprotection, reduction of neuroinflammation and in improved motor function^40^.

Physiological brain ageing is accompanied by the appearance of specific histopathological and molecular hallmarks of neuroinflammation. For example, the baseline production of pro-inflammatory cytokines like IL-1beta, TNF and IL-6 is increased and microglia have shortened and less motile processes and an enlarged cell soma in the brains of aged rodents^2^,^3^,^4^,^5^. Further, aged microglia are discussed to be impaired in their most important effector function, that is, phagocytosis^5^, which is for example illustrated by an age-dependent accumulation of CD68 immunoreactive phagosomal/lysosomal vesicles^6^,^7^. Here, we demonstrate that montelukast reduced age-associated neuroinflammation, in particular microglia activation. Montelukast treatment reduced the soma size of microglia in old rats, suggesting a less reactive phenotype. Interestingly, montelukast also reduced the size of CD68 immunoreactive particles in these aged microglia. Although the precise function of CD68 is not well understood^7^, its accumulation in the aged brain could be interpreted in the sense that aged microglia either have a higher rate of phagocytic uptake or a reduced capacity to process the incorporated material, and importantly, montelukast might modulate such activities.

So far, the beneficial functional effects of montelukast in animal models of neurological diseases have been attributed almost exclusively to its anti-neuroinflammatory action. For example, montelukast prevented the release of the pro-inflammatory cytokines IL-1beta and TNF in BV-2 microglia cells and in primary microglia cultures^41^. Also, montelukast reduced the Abeta1–42 injection-induced elevation of pro-inflammatory signalling mediators and cytokines such as NF-kappaB p65, TNF-alpha and IL-1beta *in vivo*^38^. In the present study, we reveal that montelukast elevated the expression of the anti-inflammatory cytokine TGF-beta1. Moreover, it reduced the expression levels of NOS2, a pro-inflammatory enzyme that is increased in the ageing CNS^5^, and of the chemokine and peripheral monocyte/macrophage chemoattractant CCL2, which has been identified in a proteome screen as a blood-derived ageing factor^7^. Interestingly enough, these inflammatory markers were reduced to levels even lower than those of untreated controls or of cells exposed to montelukast alone. This suggests that when cells are co-stimulated simultaneously, as in the present experiments, with both LTD4 and montelukast, montelukast does not merely antagonize the activation induced by LTD4, but very likely tunes the inflammatory systems to differential responses. This, in turn, indicates that montelukast ‘primes' the system towards an anti-inflammatory phenotype, and that it does so preferentially in the presence of pro-inflammatory signals suggesting a rather specific protective spatio-temporal effect only where and when inflammation is activated.

Alterations of the peripheral innate and adaptive immune system during ageing are well documented^42^, and there is accumulating evidence that the bidirectional crosstalk between the peripheral immune system and the CNS plays a decisive role in shaping brain functions^43^. Age-associated impairments of the peripheral immune system are under discussion to alter the highly coordinated interactions between the immune system and the brain, and to contribute to age-related cognitive declines^42^. Since montelukast was originally developed to inhibit peripheral inflammatory reactions in asthmatic patients, and has been further reported to modulate innate immune cell functions in the periphery, it is very likely that this drug might, besides its direct effects on CNS microglia activity, exert a number of anti-inflammatory responses in the CNS indirectly by modulating the peripheral immune system, for example by downregulating CCL2 in peripheral organs.

Besides its anti-inflammatory action, montelukast stabilizes the BBB, as suggested by the claudin-5 immunohistochemistry illustrated in the present study. This is further supported by the literature, as montelukast reduced the infiltration of inflammatory cells and BBB permeability in the experimental autoimmune encephalomyelitis animal model of multiple sclerosis^44^, decreased pentylenetetrazol-induced BBB dysfunction^25^, and reduced BBB permeability in an animal model of traumatic brain injury^45^. Considering the potential of montelukast to restore the BBB, it is even more important to emphasize that montelukast was detected in the brain and CSF after oral administration.

Here, we are demonstrating for the first time that montelukast promotes hippocampal neurogenesis, in particular progenitor cell proliferation, which results in an increased number of newly generated neurons. The effect on neurogenesis was, like the anti-inflammatory activity, specific for the old rats. Thus, montelukast might stimulate neural progenitor proliferation only in situations in which neurogenesis is compromised. Montelukast might liberate progenitors from age-associated inhibitory mechanisms, which most likely include elevated levels of leukotrienes. Obviously, the extrapolation of these results from normal ageing to neurodegenerative diseases is intriguing, and some of the beneficial effects of montelukast in animal models of neurodegeneration might well be attributed to enhanced neurogenesis. The fact that montelukast did not stimulate cell proliferation in the young animals is an important safety feature, as a hyperstimulation of neural progenitors might increase the risk to generate brain tumours.

An important issue to address is the mode of action of montelukast and if the drug-induced improvement in cognition is mediated through elevation of neurogenesis. Regression and correlation analyses suggested a causal link between cognition and neurogenesis in the old rats, and montelukast even fostered the correlation between neurogenesis and cognition in these animals. In contrast, learning success showed no correlation with the anti-inflammatory effects of montelukast. Thus, the montelukast-induced elevated neurogenesis rather than the anti-inflammatory action of montelukast might be the prime causal factor related to the improved cognition in old rats. This, however, requires further experimental evidence, for example through genetic, physical or pharmacological modulation or inhibition of adult hippocampal neurogenesis. Nevertheless, a correlation between cognitive performance and DG proliferation has already been reported previously in aged rodents^46^. Moreover, pharmacologic stimulation of neurogenesis by counteracting age-associated inhibitors of neurogenesis has been shown to improve cognitive functions in ageing^47^.

In general, a clear dissection between neurogenesis- and neuroinflammation-mediated effects on cognition is not straightforward as neurogenesis and neuroinflammation strongly influence each other. For example neural progenitors induce microglia proliferation and activation^48^, and vice versa, microglia regulate adult hippocampal neurogenesis^49^. We still lack detailed knowledge on the exact interrelations between specific patterns of neuroinflammation and neurogenesis. Pharmacological modulation of neuroinflammation with minocycline altered hippocampal inflammatory cytokine gene expression and significantly improved learning in the water maze paradigm in aged mice, but did not change the rate of neurogenesis in these animals, while it elevated neurogenesis without affecting cognition in young animals^50^. Hence, the minocycline-induced effects on cognition in the aged animals appear to be independent of adult hippocampal neurogenesis modulation. This further suggests that minocycline and montelukast, although both being anti-inflammatory agents, might address different types of neuroinflammation with specific consequences on neuroprotection and regenerative responses such as neurogenesis.

As one of the potential mechanisms of montelukast action, we identified elevated leukotriene production in the brains of old rats and elderly humans, and the montelukast targets CysLTR1 and GPR17 being expressed in the neurogenic niche. Indeed, montelukast might interact with CysLTR1 and with GPR17 in the brain, as we detected montelukast after oral delivery in the brain parenchyma and in the CSF. Since GPR17 is, besides being expressed in oligodendroglia, also expressed in the neuronal lineage, and since genetically mediated reduction of GPR17 (either through deletion of FoxO1,3,4 or through direct knockout of the *GPR17* gene) increased the neural progenitor proliferation *in vitro*, we suggest that the effects of montelukast on neurogenesis and cognition are most likely mediated through inhibition of GPR17. Indeed, FoxO1,3,4 as well as GPR17 knockout neurospheres did not respond to montelukast in terms of cell proliferation, and responsiveness in FoxO1,3,4 knockout cells was rescued through viral mediated expression of FoxO1, which also restored GPR17 expression. FoxO plays a crucial role in regulating neural stem cell proliferation and self-renewal, as FoxO1,3,4 knockout animals show increased brain size and proliferation of neural progenitor cells during early postnatal life leading to an exhaustion of the stem cell pool and to low neurogenesis in adulthood^51^. This has been attributed to the effects of a number of FoxO targets, such as genes that cooperatively act as soluble inhibitors of the Wnt pathway to repress Wnt signalling in neural stem cells^51^. GPR17 signalling might be one of such FoxO regulated pathways that are involved in neural stem and progenitor modulation, in particular in the aged brain. Targeting GPR17 might further be relevant for acute CNS lesions such as spinal cord injury, since GPR17 has been identified in spinal cord ependymal stem cell like cells, and the *in vivo* knockdown of GPR17 by antisense oligonucleotides in a spinal cord injury model reduced tissue damage and motor deficits^52^.

What are the upstream signalling components relevant for the observed montelukast effects? Montelukast is an antagonist of CysLTR1 and of GPR17, favouring the hypothesis that leukotrienes are the relevant ligands that cause neuroinflammation and reduce neurogenesis in the brains of old animals. The leukotriene signalling pathway is currently under discussion to contribute to brain inflammation associated with age-related dementia and neurodegenerative diseases^18^,^20^. In the CNS, 5-LOX mRNA levels are elevated in the hippocampus in an Alzheimer's disease animal model^53^ and in the hippocampus of Alzheimer's disease patients^54^. In addition, the concentrations of 5-LOX transcripts and of leukotrienes are increased specifically in the hippocampus of old rodents, as shown in the present work and by others^21^,^55^. Furthermore, intracerebral infusions of LTD4 in adult mice lead to an accumulation of Aβ 1–40 and Aβ 1–42 in the hippocampus and cortex as well as to memory deficits, which could be inhibited by pre-treatment with the CysLT1R antagonist pranlukast^20^. Although leukotrienes mediate and/or exaggerate pathogenesis in the CNS, it appears that these effects of leukotrienes are context-dependent. For example, leukotrienes alone did not induce neuronal cell death in rat cortical neurons *in vitro*^56^.

Besides leukotrienes, other ligands and agonists for GPR17 have been described, which might well contribute to the activation of the receptor in ageing and in neurodegenerative diseases. For example, UDP-glucose, which is elevated in the brain after damage, has been described as GPR17 agonist^52^, and UDP-glucose stimulates oligodendroglial progenitor migration and differentiation by activating GPR17 (ref. 57). Also, oxysterols, which act as inflammatory mediators, have recently been identified as potential GPR17 ligands and agonists^58^. Nevertheless, more recent studies using GPR17 overexpression in different cell lines reported that neither UDP-glucose nor leukotrienes were ligands for GPR17 (refs 59, 60), leaving the question of the physiologic ligands of GPR17 still open for future discoveries.

Similarly, the exact downstream signalling components that mediate the beneficial effects of montelukast in the old rats are not fully deciphered at present, and leukotriene receptor-independent effects cannot be excluded. In this context, Yagami *et al.*^56^ suggested that voltage-gated calcium channels mediated neuroprotective effects of the leukotriene receptor antagonist pranlukast in an *in vitro* model of brain ischaemia. Moreover, a network-based interference approach has recently identified montelukast as a potential inhibitor of dipeptidyl peptidase-IV, a proteolytic enzyme and target of a number of approved anti-diabetes drugs which increase the release of insulin^61^. Of interest in the context of the present study, such anti-diabetic drug treatment could attenuate pathogenesis in animal models of Alzheimer's disease^62^, Parkinson's disease^62^ and in an animal model of multiple sclerosis^63^.

In summary, we demonstrate for the first time the possibility of using montelukast to functionally rejuvenate the aged but otherwise healthy brain. Oral treatment with montelukast restored learning and memory in old rats, which was significantly impaired in comparison to young animals. Considering the various beneficial effects of montelukast on CNS functions in different animal models, this illustrates that leukotriene receptors and their underlying signalling mechanisms might contribute to the development of many neurological deficits, and that montelukast, by targeting these mechanisms, might be able to modulate and to improve a number of neurological functions in various CNS diseases.

## Methods

Methods that were required to generate Supplementary Data are provided in the Supplementary Material.

### Animals

For the *in vivo* experiments, young (4 months) and old (20 months) male transgenic *DCX-DsRed2* Fisher 344 rats were used. They express the fluorescent reporter DsRed2 under control of the neuronal precursor cell-specific promoter of the *doublecortin (DCX)* gene and were originally generated at the transgenic mouse facility of the University of Heidelberg, Germany, and previously described^64^. For all other studies on rat tissue, young (4 months) and old (20 months) male wild-type Fisher 344 rats (Charles River, Germany) were used. All rats were bred and housed in the animal facility of the PMU Salzburg under standard conditions of a 12 h light/dark cycle with food and water *ad libitum*. All animal care and use was in accordance with the European Community Council Directive (86/609/EEC) and approved by the Federal State Land Salzburg, Austria (20901-TVG/40/6–2010). The numbers of rats used to result in statistically significant differences were calculated using G*Power 3.1.7 software. Standard power analyses with *α*=0.05 and a power of 0.8 were performed to calculate power and sample sizes. For neurosphere culture experiments, *FoxO1/3/4*^*fl*^ mice^65^ (P56, male, *N*=6 animals) were obtained from Ronald A. DePinho (University of Texas MD Anderson Cancer Center, Houston, US) and *GPR17*^*−/− GFP*^ mice^66^ (2 months old, male, *N*=10 animals) were provided by Douglas Fields (NICHD, Bethesda, USA). Mouse tissue was collected in conformity with the Austrian Federal Law for experiments on living animals (TVG2012§2).

### Montelukast treatment and BrdU injections

Montelukast sodium powder (Sigma), dissolved in ethanol for maximum solubility and then further diluted (1:9 ratio) with a 0.9% saline (NaCl) solution, was administered daily per oral gavage (p.o.) at a dose of 10 mg kg^−1^ of body weight for 42 days to young adult (4 months) and old (20 months) rats (*N*=7–10 rats/group). Age-matched control rats received oral gavage of the vehicle solution (10% ethanol in 0.9% NaCl). For the analysis of cell survival and cell differentiation, all rats received intra-peritoneal injections of BrdU (Sigma) at 50 mg kg^−1^ of body weight dissolved in 0.9% saline solution on days 14, 15, 16 and 17. Weight and general appearance of the rats was recorded daily during the course of the experiment. Criteria for early termination of treatment were obvious signs of pain, apathy, and loss of weight more than 15%. During the course of this experiment, one vehicle-treated rat (20 months old) was killed because of a weight loss of more than 15% for unknown reasons.

### Behavioural tests

28 days after starting the montelukast treatment, several standardised behavioural tests were carried out. All parameters analysed were recorded via the use of the video tracking software Ethovision 9.0 (Noldus Information Technology).

In the open field test (day 29 after the first montelukast administration), spontaneous changes in general locomotor activity were detected. The open field was a black circular plastic plate with a diameter of 1 m and a 5 cm high wall, set in the middle of the testing room. Before a new trial was begun, the open field was cleaned with 70% ethanol. Each rat was placed into the centre of the plate and was allowed to explore the apparatus for 5 min. The total distance moved was recorded.

The elevated plus maze (performed on day 30 after the first montelukast administration) was used to test anxiety of the rats. The wooden maze had four arms of the same dimensions (50 × 10 cm) and was situated 50 cm above the floor. Two opposite arms were enclosed by 20 cm high opaque walls (closed arms). To reduce olfactory influences, the maze was cleaned with 70% ethanol after every trial. The rats were allowed to perform trials with 10 min of free exploration starting in the central intersection facing an open arm. The camera software recorded the time spent in the closed arms, in the open arms, and in the centre zone, and the overall distance moved.

The forced swim test was performed on day 33 to analyse depressive-like behaviour. Rats were forced to swim for 10 min in a square plastic tank (40 cm side length) filled to a depth of 30 cm with tap water (20±1 °C). During the forced swimming session, the behaviour was recorded by a video system and scored by a trained observer, quantifying absolute time measurements. The behaviour of the animals was assigned to one of the three following behavioural categories: (1) ‘struggling', defined as movements during which the forelimbs broke the surface of water; (2) ‘swimming', defined as movement of the animal induced by movements of the fore and hind limbs without breaking the water surface; and (3) ‘immobility time', defined as the behaviour during which the animal used limb movement just to keep its equilibrium without any movement of the trunk. After the 10 min swimming session, animals were gently dried using a towel and returned to their home cage.

The Morris water maze test (days 35–41) was used to assess the ability of spatial learning and memory. The apparatus consisted of a circular swimming pool built of black plastic (170 cm diameter, 30 cm height), filled with 21 °C±1 °C tempered water. The tank was virtually divided into four equal quadrants, with a submerged hidden 10 × 10 cm fibreglass platform placed 3 cm below the water surface in the middle of the target quadrant. The position of the platform was kept unaltered throughout the learning sessions. In the testing room, several big black cue symbols were put on each wall for spatial orientation. The water maze task was carried out twice a day for 5 consecutive days. One day before starting the learning experiment (day 0), each rat was put into the water and was allowed to locate the submerged platform for 60 s. If the animal failed to find the platform within the 60 s, it was guided onto the platform and allowed to remain there for 10 s. For the learning tasks on days 1–5, each rat was put into the water at one of four starting positions, the sequence of which being selected randomly. In each trial, a ceiling time of 60 s to find the platform was defined. The escape latency time to locate the hidden platform and the distance moved during the trial were recorded with the camera software as indices of spatial learning. On day 6, after the learning phase of the experiment, a probe trial was performed. Here, the platform was removed and each animal was allowed to explore the pool for 60 s. From the probe trial, several parameters were recorded as indices of memory: time spent in platform quadrant, number of crossings of the former platform location, number of crossings of ‘zone P' (a defined circular area with 20 cm diameter enclosing the former platform location) and time spent in zone P.

A learning index was calculated for every rat based on the individual learning curve obtained in the water maze spatial learning paradigm. For this, the value of the area under the curve between days 0 and 5 was calculated and subtracted from 300, which represents the value for the worst possible performance. Thus, high values indicate successful learning, while low learning indices illustrate poor spatial learning.

For the object location memory test, a test of hippocampus-dependent memory functions, a different cohort of old (20 months) male Fisher 344 rats was used. Animals received montelukast and vehicle administration as well as BrdU injections as described above (*N*=5 per group), and on days 35–39 of montelukast treatment, the object location memory test was performed. Prior to training, rats habituated to the experimental apparatus (a 70 × 70 × 40 cm open top plastic box) for 5 min day^−1^ in the absence of objects for 3 days. During the training period, rats were placed into the box with two identical objects (beverage cans; 13.5 × 5.2 cm) and allowed to explore for 15 min. During the long-term retention test, that is, 24 h following the training period, one of the object locations was shifted and the rats were allowed to explore the experimental apparatus for 10 min. Exploration was scored when a rat's head was oriented toward the object within a distance of 2 cm or when the nose was touching the object. The relative exploration time (*t*) of the newly positioned object was recorded and expressed as a novelty index (*NI*=*t*_novel_/(*t*_novel_+*t*_familiar_) × 100%).

### Perfusion and tissue processing

On day 42, all rats were deeply anaesthetized using a ketamine (20.38 mg ml^−1^), xylazine (5.38 mg ml^−1^) and acepromazine (0.29 mg ml^−1^) mixture. Transcardial perfusion was performed with 0.9% NaCl solution, followed by a 4% paraformaldehyde, 0.1 M sodium phosphate solution (pH 7.4). The brains were dissected and post-fixed in the paraformaldehyde solution overnight at 4 °C. Tissues were then cryoprotected in a 30% sucrose solution. Brains were cut into 40 μm saggital sections using a sliding microtome on dry ice. Sections were stored at −20 °C in cryoprotectant solution (ethylene glycol, glycerol, 0.1 M phosphate buffer pH 7.4, 1:1:2 by volume).

### Immunohistochemistry

Free-floating sections were treated with 0.6% H_2_O_2_ in tris-buffered saline (TBS: 0.15 M NaCl, 0.1 M Tris-HCl, pH 7.5) for 30 min, followed by three washes with TBS. For immunological detection of PCNA and BrdU, sections were incubated in 0.3 M NaCl/30 mM citrate buffer (pH7.0)/ 50% formamide at 65 °C for 2 h, rinsed in 0.3 M NaCl/30 mM citrate buffer (pH 7.0), incubated in 2 N HCl at 37 °C for 30 min, rinsed in 0.1 M borate buffer (pH 8.5) for 10 min and again rinsed in TBS.

All sections were blocked with a solution composed of TBS, 0.1% Triton X-100, 1% bovine serum albumin (BSA), and 0.2% teleostean gelatin (Sigma) for 1 h. This buffer was also used during the incubation with primary antibodies, which were applied overnight at 4 °C. For chromogenic immunodetection, the sections were washed extensively and further incubated for 1 h with a biotin-conjugated species-specific secondary antibody. Sections were then incubated for 1 h in a peroxidase-avidin complex solution (Vectastain Elite ABC kit; Vector Laboratories). The peroxidase activity of immune complexes was revealed with a solution of TBS containing 0.25 mg ml^−1^ 3,3 diaminobenzidine (Vector Laboratories), 0.01% H_2_O_2_, and 0.04% NiCl_2_. The following antibodies and final dilutions were used. Primary antibodies: rat anti-BrdU (1:500, BU1/75, Serotec), rabbit anti-CD68 (1:300, ab125212, Abcam), rabbit anti-CysLTR1 (1:100, SP4109P, Acris), rabbit anti-Doublecortin (1:300, 4604, Cell Signaling), goat anti-Doublecortin (1:200, sc-8066, Santa Cruz), mouse anti-5-LOX (1:100, 610695, BD Transduction Laboratories), guinea pig anti-GFAP (1:500, GP52, Progen), rabbit anti-GPR17 (1:400, 10136, Cayman), rabbit anti-Iba1 (1:300, 019–19741, Wako), goat anti-Iba1 (1:250, ab107159, Abcam), mouse anti-NeuN (1:500, A60, Millipore), goat anti-Olig2 (1:300, AF2418, R&D Systems), goat anti-PCNA (1:250, C-20, Santa Cruz), goat anti-Sox2 (1:500, Y-17, Santa Cruz). Secondary antibodies: donkey anti-goat, -mouse, -rabbit Alexa 488, donkey anti-guinea pig Alexa 568, donkey anti-goat, -guinea pig, -mouse, -rabbit Alexa 647 (all 1:1000, Invitrogen), donkey anti-rat Cy5, donkey anti-mouse, -goat, -rabbit, -rat biotinylated (all 1:500, Jackson Immuno Research). Nuclear counterstaining was performed with 4', 6'-diamidino-2- phenylindole dihydrochloride hydrate at 0.25 μg μl^−1^ (DAPI; Sigma). Secondary antibody controls, in which the first antibody was omitted, were performed for all experiments to ensure specificity of the antibody staining. Chromogenic immunodetection was photodocumented using a Zeiss Axioplan microscope (Zeiss,) equipped with the Zeiss AxioVision imaging system. Epifluorescence observation was performed on a confocal scanning laser microscope (LSM 710, Zeiss) with LSM software (ZEN 2011).

### Quantitative analysis of immunohistological data

For quantification of different cell types in the DG, analyses were performed blinded on coded slides. Every tenth section (400 μm interval) of one hemisphere was selected from each animal and processed for immunohistochemistry. To analyse cell proliferation and survival, PCNA- or BrdU-labelled cells within the DG were counted on a Zeiss Axioplan microscope. The reference volume of the DG was determined by tracing the DG area on each analysed section. To estimate the numbers of Sox2^+^, DCX^+^, BrdU^+^/NeuN^+^, BrdU^+^/GFAP^+^, Iba1^+^, Iba1^+^/PCNA^+^ and Iba1^+^/CD68^+^ cells in the DG, four randomly selected visual fields per animal were photographed using a confocal scanning laser microscope (LSM 710, Zeiss) with LSM software (ZEN 2011). From these representative pictures, all positively labelled cells were counted. The corresponding tissue area was measured and multiplied by 40 μm to obtain the tissue volume. To assess cell densities, the total number of cells counted was divided by the sample volume and represented as cells per mm^3^. Because of the low level of neurogenesis in old animals, in case the total number of BrdU-positive cells remained below 50, every BrdU-labelled cell detected was examined. For assessment of possible alterations in the activation state of microglia within the hippocampus, the soma sizes of the Iba1^+^cells within the DG were determined using the ‘Analyze Particles' function of ImageJ 1.45 s (ImageJ website: http://imagej.nih.gov/ij/). For each animal at least 40 microglial cells were assessed. The ‘Analyze Particles' function of ImageJ was also used to analyse the mean size of CD68^+^ particles in four randomly selected visual fields (maximum projection of the *z*-stack across the whole section). For each animal, at least 120 particles were measured.

### Human post-mortem brain tissue

Human hippocampal tissue sections from autopsy samples of young adult (<35 years, *N*=5) and elderly (>60 years, *N*=5) humans with a post-mortem interval <24 h were used. Human post-mortem tissue was obtained from the collection of the Department of Neuropathology of the University Hospital Erlangen (Germany). Written informed consent was obtained from the patients' next of kin. The use of these specimen for scientific purposes was in accordance with institutional ethical guidelines and was approved by the ethics committee of the University of Erlangen (Germany). All samples used were obtained from individuals without any neurological or psychiatric diagnoses. After tissue extraction, the brain samples were fixed overnight in 10% formalin and subsequently processed into liquid paraffin. All tissue samples were cut at 5 μm on a rotation microtome and stored at 4 °C.

### Formalin-fixed paraffin-embedded immunohistochemistry

Formalin-fixed paraffin-embedded human tissue sections were deparaffinized by incubation in xylene, rehydrated by a graded series of ethanol and rinsed in distilled water for 5 min. For antigen retrieval, the slides were steamed in 0.01 M sodium citrate buffer, pH 6.0 at 100 °C for 20 min, followed by three washes in TBS. Endogenous peroxidases were quenched with 0.3% hydrogen peroxide for 20 min. Sections were blocked with a solution composed of TBS, 0.1% Triton X-100, 1% BSA and 0.2% teleostean gelatin (Sigma) for 20 min, and incubated overnight at 4 °C with a rabbit anti-5-LOX antibody (1:50; 610695, BD Pharmingen) diluted in the blocking solution. After incubation, the sections were washed in TBS, incubated with a biotinylated rabbit anti-mouse antibody (1:500, Jackson Immuno Research) for 1 h, washed in TBS, and incubated for 1 h in a peroxidase-avidin complex solution (Vectastain Elite ABC kit; Vector Laboratories). The peroxidase activity of immune complexes was revealed with a solution of TBS containing 0.25 mg ml^−1^ 3,3 diaminobenzidine (Vector Laboratories), 0.01% H_2_O_2_, and 0.04% NiCl_2_. Sections were mounted with Neo-Mount (Merck).

### RNA extraction and quantification of 5-LOX mRNA

To detect 5-LOX mRNA levels in different brain regions of young and old rats, total RNA was extracted from rat hippocampus, subventricular zone and cortex from 4-month- and 20-month-old Fisher 344 wild-type rats. Animals were decapitated and the tissues of interest were dissected. Brain samples were homogenized in 1 ml of Trizol (TRI Reagent; Sigma). For phase separation, 200 μl of 1-bromo-3-chloropropane were added, vortexed and centrifuged (15 min at 12,000 g). After transferring the aqueous phase into a new tube and adding 350 μl of ethanol, RNA extraction was performed using the QIAGEN RNEasy Mini Kit (Qiagen) and cDNA was synthesized using Promega reverse transcription Kit (Promega).

Quantitative 5-LOX gene expression analysis was performed by the TaqMan gene expression assay kit (Catalogue number 4369514, Applied Biosystems) and a specific validated 5-LOX gene expression assay (Catalogue number Rn00689111_m1, Applied Biosystems). GAPDH was used as endogenous control gene for rat 5-LOX quantification. The following temperature profile was used: activation of polymerase 95 °C, 10 min; 40 cycles of denaturing 95 °C, 15 s, and annealing/extension 60 °C, 60 s. Data were obtained with a Rotor-Gene 6000R Corbett Research (geneXpress) and analysed by delta delta Ct method^67^.

### BV-2 cell culture experiments

Cells from the murine microglial cell line BV-2 (ref. 68) were originally obtained from Banca Biologica e Cell Factory, IRCCS Azienda Ospedaliera Universitaria San Martino, Genua, Italy, and tested for mycoplasma contamination (Myco Alert, Lonza) before further use. Cells were maintained in Dulbecco's Modified Eagle's Medium (Life Technologies) supplemented with 10% fetal bovine serum and antibiotics (penicillin 100 U ml^−1^, streptomycin 100 U ml^−1^, HVD Life Sciences) under standard culture conditions (95% relative humidity with 5% CO_2_ at 37 °C). For RNA expression studies, subconfluent BV-2 cells were treated for 24 h either with 100 nM LTD4 (Sanova Pharma), 15 μM montelukast (Sigma), or with a combination of LTD4 and montelukast, in which the cells were pretreated with 15 μM montelukast (Sigma) for 15 min before 100 nM LTD4 were added. The controls received only the vehicle solution (ethanol) for 24 h.

Quantitative gene expression analyses were performed using TaqMan PCR with reverse transcription technology. RNA was isolated with the RNeasy Mini Plus Kit (Qiagen) and transcribed to cDNA using the Reverse Transcription System (Promega) according to the manufacturer's instructions. Technical duplicates containing 10 ng of reverse transcribed RNA were amplified with the GoTAQ Probe qPCR Master Mix (Promega) using a two-step cycling protocol (95 °C for 15 s, 60 °C for 60 s; 40 cycles, Bio-Rad CFX 96 Cycler). The following gene expression assays were employed: Arg-1 (Mm00475988_m1, Applied Biosystems), CCL2 (Mm.PT.56a.42151692, Integrated DNA Technologies), NOS-2 (Mm00440485_m1, Applied Biosystems), TGFb1 (Mm.PT.56a.11254750, Integrated DNA Technologies), TNF (Mm00443258_m1, Applied Biosystems) as well as the following validated housekeepers: HPRT1 (Mm00446968_m1, Applied Biosystems), PSMD4 (Mm.PT.56.13046188, Integrated DNA Technologies), SDHA (Mm01352366_m1, Applied Biosystems). Quantification analyses were performed with qBase Plus (Biogazelle) using geNorm algorithms for multi-reference gene normalization followed by normalization to control conditions.

### *FoxO1/3/4* ^*fl/fl*^ mouse neurospheres

A mouse line with floxed alleles for *FoxO1*, *FoxO3* and *FoxO4* in FVB/N background was used (*FoxO1/3/4*^*fl/fl*^ mice) for the preparation of neural stem cells. Six male *FoxO1/3/4*^*fl/fl*^ mice (P56) were killed via cervical dislocation and brains were kept in ice-cold PBS. The subependymal zone was isolated under binocular microscope and kept further in ice-cold PBS. Neural stem cells were isolated and cultured as described before^69^. Briefly, cells were resuspended in Neurobasal (NB) medium (Gibco BRL) supplemented with B27 (Gibco BRL), 2 mM L-glutamine (PAN), 100 U/ml penicillin/0.1 mg l^−1^ streptomycin (PAN), hereafter referred to as NB/B27. For maintenance and expansion of the cultures, the NB/B27 was further supplemented with 2 μg/ ml heparin (Sigma), 20 ng/ml FGF-2 (R&D Systems) and 20 ng/ml EGF (R&D Systems) (NB-A). Cultures were maintained in T-25 culture flasks at 37 °C in a humidified incubator with 5% CO_2_. Passages 2–10 were used througout this study. For passaging these cells, the culture medium containing floating neurospheres was collected in a 15- ml Falcon tube and centrifuged at 120 × g for 5 min. The pellet was resuspended in 200 μl of 1% Accutase (PAA) and triturated approximately 10 times using a pipette. Dissociated cells were centrifuged at 120 × g for 5 min, resuspended and reseeded.

For retroviral transduction of neural stem cells, mouse neurospheres from *FoxO1/3/4*^*fl/fl*^ mice were passaged and dissociated into single cells as described above. Cells were counted and resuspended in 2 ml culture medium. Virus particles of a GFP/Cre-Recombinase-expressing retrovirus or an only GFP-expressing retrovirus corresponding to the number of counted cells were added and the cells were incubated at 37 °C for 30 min until the virus particles were inactive. The transduction was controlled after 4–7 days via fluorescence microscopy. The cells were FACS sorted to obtain homogenous populations. Knockout of the cells transduced with the GFP/Cre-Recombinase-expressing retrovirus was controlled via genotyping. In the following, cells from *FoxO1/3/4*^*fl/fl*^ mice that have been successfully transduced with the GFP/Cre-Recombinase-expressing retrovirus and that show a FoxO1/3/4 knockout, are termed ‘*FoxO1/3/4*^*−/−*^' or ‘*FoxO1/3/4 KO*'.

### Overexpression of *FoxO1* and rescue of *FoxO1/3/4 KO*

Both control cells, which were transduced with a GFP-expressing retrovirus, and *FoxO1/3/4 KO* cells, which had been transduced with a GFP/Cre-Recombinase-expressing retrovirus, were transduced additionally with a lentivirus coding for a dominant active form of FoxO1 (Lenti-FoxO1-ADA) as described above. This lentivirus was constructed by cloning the FoxO1-ADA of the pCMV-FoxO1-ADA^70^ into the expression cassette of the pLL3.7 lentivirus (Addgene).

For quantitative gene expression analyses, total RNA was isolated from control and *FoxO1/3/4 KO* neurospheres (with and without FoxO1 rescue) by using the RNeasy Mini-Kit (Quiagen) according to the manufacturer's protocol. Possible genomic DNA contamination was eliminated by on-column DNAse treatment using the RNAse-free DNAse-Set according to the manufacturer's protocol. cDNA was transcribed using the Fermentas RevertAid First Strand cDNA Synthesis Kit (Thermo Fisher Scientific) according to the manufacturer's instructions.

Quantitative PCR with reverse transcription was performed using the StepOnePlusTM Real-Time PCR System. Brilliant II Fast SYBR Green quantitative PCR (qPCR) Master Mix was used for PCR reactions according to the manufacturer's protocol. qPCR primers were designed using the software's Primer3 ( http://primer3.sourceforge.net) and NetPrimer ( http://www.premierbiosoft.com). Amplicon sizes ranged from 100–250 bp. Suitability of qPCR primer was analysed by evaluation of melting curves and by determination of the efficiency via a standard curve. For quantitative expression analysis, the delta delta CT method^67^ was applied to determine the relative quantity of target sequence using a reference sample (control) and an endogenous control target sequence. RPL27 was used as endogenous control target. Primers for qPCR were as follows: RPL27: forward, CCTGGATAAACTGTGGACATTGG; reverse, TGTAGTAGCCTGATCGAACAACA; GPR17: forward, CGACTCACTGGCTTCCTCTT; reverse, GCCAGGTGAGCATAGAGAGG, CysLTR1: Forward, CCTCTCCGTGTGGTCTATTATGT; reverse: ACCGGAAAAAGCTCATGGCT.

### *GPR17* ^*−/−*^ neurospheres

Neurospheres were obtained from 2-month-old male *GPR17*^*−/− GFP*^ mice (*N*=10), in which the entire GPR17 coding exon is replaced by an h2bGFP/neo cassette via homologous recombination^66^. Age-matched *C57BL/6* wild-type mice (*N*=10) were used as controls. Neurospheres were isolated and cultured as described for *FoxO1/3/4*^*fl/fl*^ mouse neurospheres above. Passage numbers 2–8 were used throughout this study.

### Single-cell cultures—single-cell neurosphere assay

To culture a neurosphere out of a single neural stem cell, neurospheres were diluted as 1 cell per 25 μl neurosphere media with 20 ng ml^−1^ growth factors (EGF and FGF-2) with or without montelukast (10 μM, Sigma), and seeded into the wells of a 60-well Nunc MicroWell MiniTray (Thermo Scientific) plate. After seeding the cells, the wells were examined under a binocular microscope/fluorescent microscope and those wells that contained one or two cells were marked. After 7 days, the marked miniwells were examined again under a binocular microscope/fluorescent microscope and the number of formed neurospheres was counted.

### Neurosphere bulk assay

A total of 5 × 10^4^ cells were seeded in a 25-cm^2^ culture flask in 5 ml medium under proliferative conditions (with 20 ng ml^−1^ EGF and FGF-2). Montelukast was added to a final molarity of 10 μM on days 0, 2, 4 and 6 (control cells received medium only). After 7 days, neurospheres were harvested and dissociated into single cells. Total numbers of cells were counted and normalized to untreated control cells.

### Analysis of cell proliferation—MTS-assay

After passaging, cells were seeded in 96-well culture plates at a concentration of 5 × 10^4^ cells/ml in a volume of 100 μl NB/B27 medium under proliferative conditions (with 20 ng ml^−1^ EGF and FGF). The plates were then maintained at 37 °C in a humidified incubator (Heraeus) with 5% CO_2_ for 7 days. At days 0, 2, 4 and 6, montelukast was added to a final molarity of 10 μM to the wells (control cells received medium only). At day 7, proliferation was assessed using an MTS assay kit (CellTiter 96 AQueous One Solution Cell Proliferation Assay, Promega) according to the manufacturer's instructions. After 4 h of incubation, optical density was measured at 490 nm using a plate reader (Emax Precision Microplate Reader, Molecular Devices).

### Adult rat neurosphere cultures

2–4 month-old female Fischer 344 rats (*N*=6) (Charles River) were decapitated and hippocampi were aseptically removed and dissociated. Neurosphere cultures were obtained as for FoxO1/3/4^fl/fl^ mouse neurospheres described above. Cultures from passage numbers 2–8 were used throughout this study.

### Immunocytochemistry

For immunocytochemical analysis of BV-2 cells and mouse neurospheres from *FoxO1/3/4*^*fl/fl*^ mice, fixed cells were washed in TBS (0.15 M NaCl, 0.1 M Tris-HCl, pH 7.5), then blocked with a solution composed of TBS, 1% BSA and 0.2% Teleostean gelatin (Sigma) (fish gelatin buffer, FSGB). The same solution was used during the incubations with antibodies. Primary antibodies were applied overnight at 4 °C. Fluorochrome-conjugated species-specific secondary antibodies were used for immunodetection. The following antibodies and final dilutions were used. Primary antibodies: rabbit anti-CysLTR1 (1:500, SP4109P, Acris), chicken anti-GFP (1:1,000; GFP1020, Aves Labs) and rabbit anti-GPR17 (1:400, 10136, Cayman).Secondary antibodies: donkey anti-rabbit Alexa 568, donkey anti-chicken Alexa 647 (all 1:1,000, Invitrogen). Nuclear counterstaining was performed with 4', 6'-diamidino-2- phenylindole dihydrochloride hydrate at 0.25 μg μl^−1^ (DAPI; Sigma). Specimens were mounted on microscope slides using a Prolong Antifade kit (Molecular Probes). Epifluorescence observation was performed on a confocal scanning laser microscope (LSM 710, Zeiss,) with LSM software (ZEN 2011).

### Statistical analysis

Histological and behavioural experiments were randomized and performed blinded. Groups were unblinded at the end of each experiment before statistical analysis. Statistical analyses were performed using the GraphPad Prism 5.0 software (GraphPad Software) and IBM SPSS Statistics 20 software (IBM Corporation). Data were tested for normality using the Kolmogorov–Smirnov or the Shapiro–Wilk test, and equality of variance was confirmed using the F-test. Means between two groups were compared by the two-tailed unpaired Student's *t*-test or, in case of non-gaussian distribution, by using the two-tailed Mann–Whitney *U*-test. Data from multiple groups were analysed by one-way ANOVA, and two-way ANOVA, followed by the appropriate *post hoc* tests (as indicated in the figure legends) when necessary. Learning index correlation analyses were performed with the ‘Pearson Product Moment Correlation test'.

## Additional information

**How to cite this article:** Marschallinger, J. *et al.* Structural and functional rejuvenation of the brain in old rats by an approved anti-asthmatic drug. *Nat. Commun.* 6:8466 doi: 10.1038/ncomms9466 (2015).

## Supplementary Material

Supplementary Information Supplementary Figures 1-4 and Supplementary Methods
