# Supplementary material for: Structural and functional rejuvenation of the aged brain by an approved anti-asthmatic drug
Source: Nat Commun. 2015 Oct 27;6:8466. doi: 10.1038/ncomms9466 (PMC4639806; doi:10.1038/ncomms9466)
Supplement: Supplementary Information — Supplementary Figures 1-4 and Supplementary Methods [file ncomms9466-s1.pdf]

Supplementary Figure 1: Blood-brain barrier penetrance of systemically administered montelukast.

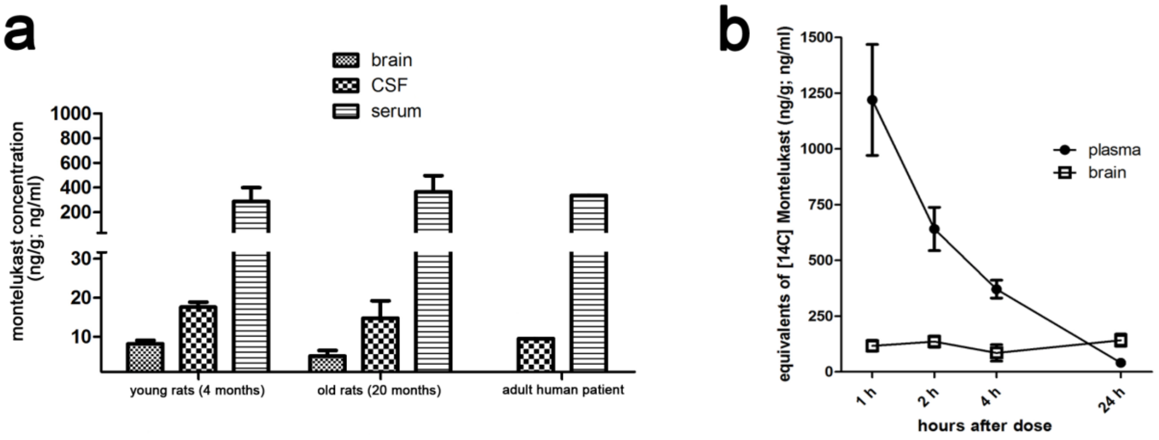

(a) Young (4 months) and old (20 months) rats received montelukast (10 mg kg^{-1} body weight) for seven days per oral gavage. One hour after the last dose, the presence of montelukast in serum, brain, and cerebrospinal fluid (CSF) was analysed by HPLC. Remarkably, irrespectively of the age, montelukast was detected in the brain and in the CSF. Most importantly, in a human subject taking 10 mg per day montelukast, the drug was detectable in the serum and in the CSF in a similar concentration as in the rats. N per group: 6 (young rats), 4 (old rats). **(b)** Re-analysis of data from the original pharmacokinetic report on montelukast[1] illustrates that one hour after a single 5 mg kg^{-1} intravenous drug administration, a substantial amount of radioactive equivalents of [C14] montelukast (brain: 117± 24 ng/g; plasma: 1220± 249 ng/ml) had reached the brain. 24 hours after montelukast injection, its levels in the brain (142± 27 ng/g) were even higher than in the plasma (41± 2ng/ml). N per group = 3.

1. FDA, Montelukast (Singulair ®, application number 20-829) (Pharmacology Review).

Supplementary Figure 2: Montelukast treatment increases expression of the tight-junction protein claudin-5 in blood vessels of old rats.

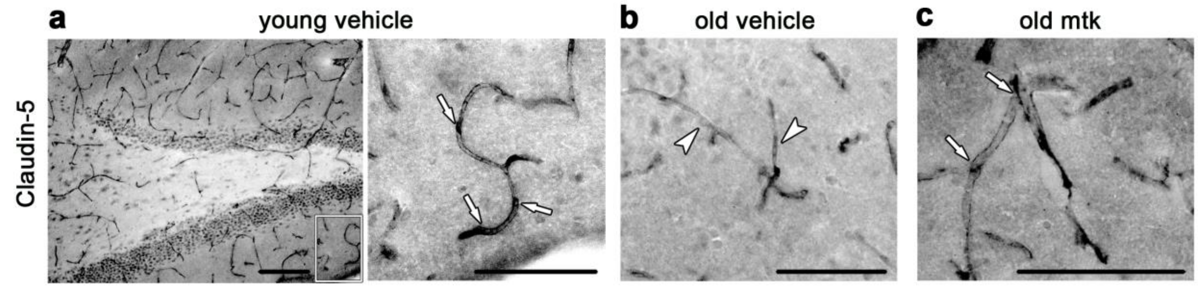

(**a**) Endothelial cells in the hippocampus of young (4 months) vehicle rats showed strong and continuous immunostaining with anti-claudin-5 antibody, indicating an intact BBB. (**b**) In contrast, claudin-5 immunostaining in endothelial cells of old (20 months) vehicle-treated rats was diffuse and weak, which points toward a disrupted BBB. (**c**) Old montelukast-treated rats (daily oral administration of 10 mg kg^{-1} montelukast for 6 weeks) showed more intense, more continuous and better-defined endothelial claudin-5 staining than age-matched vehicle controls. Arrows show strong and well-defined claudin-5 staining, arrowheads point to weak claudin-5 immunoreactivity. Scale bars: 200 µm (**a**-left panel), 100 µm (**a**-right panel,**b**,**c**). Depicted images are representative pictures for the different age- and treatment groups. 5 rats per group were analysed. *mtk* montelukast.

Supplementary Figure 3: Montelukast did neither affect synaptic density nor neuronal network activity.

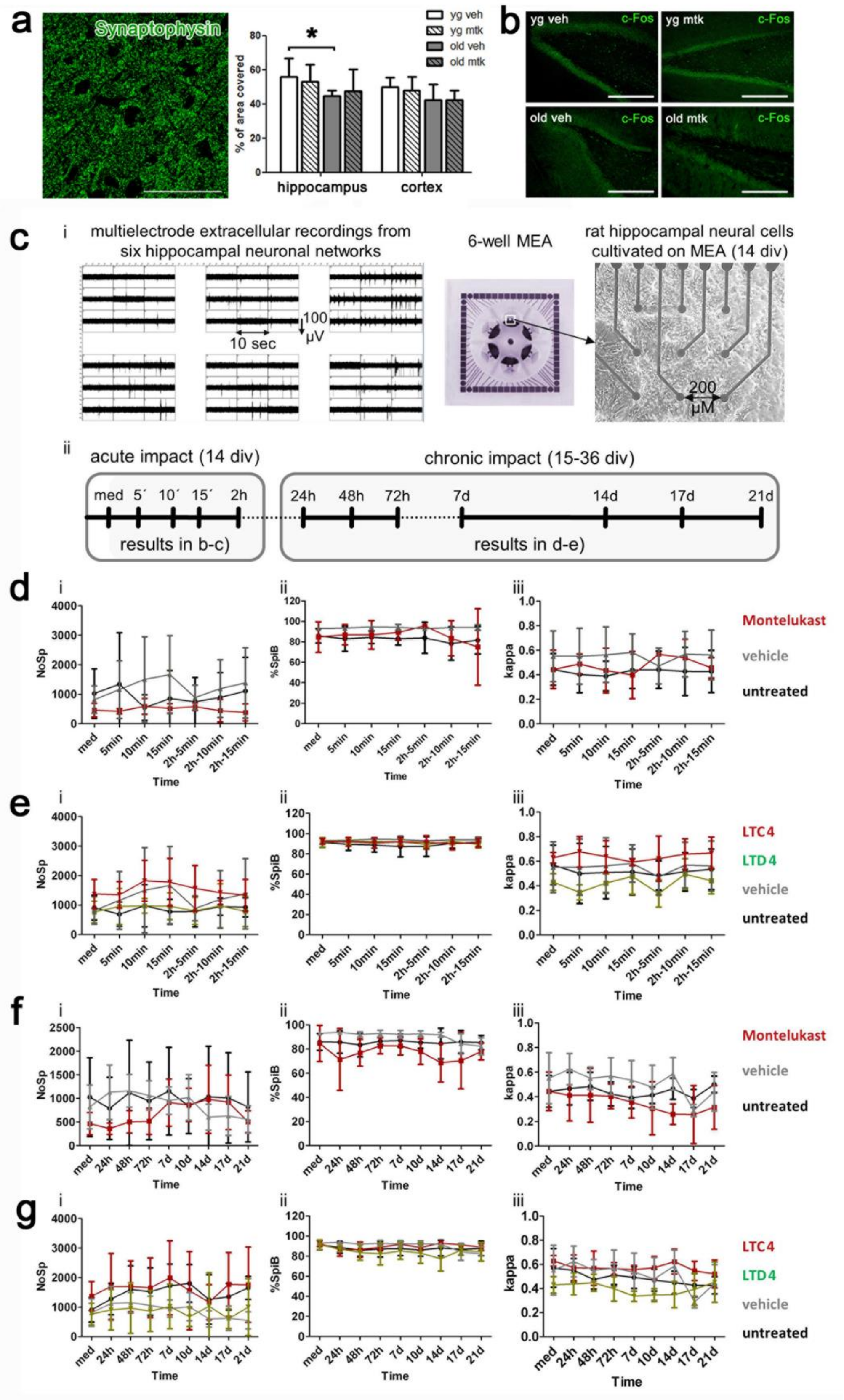

(**a**) Immunoreactive synaptophysin area was significantly reduced in old (20 months) compared to young (4 months) rats in the DG. Montelukast treatment did not change the percentages of synaptophysin$^+$ areas in young or old rats. N per group: 10 (young vehicle), 10 (young montelukast), 7 (old vehicle), 7 (old montelukast). (**b**) Amount and intensity of c-Fos expression, an immediate early gene and marker for activated neurons, was similar between age- and treatment groups in the DG (not quantitatively assessed). (**c**-**g**) Acute and chronic effects of montelukast, and of the leukotrienes LTC4 and LTD4 on network activity of rat hippocampal neuronal cultures were analysed. (**c**) Introduction of the recording device and schematic drawing of the experimental design. i) Representative neuronal network activity under medium conditions recorded from six independent hippocampal neuronal assemblies cultivated on a 6-well multielectrode array (MEA). ii) Schematic drawing of the experimental design indicates the time period (grey background) of the application of either montelukast (endc. 15 µM), LTC4, LTD4 (endc. 100 nM, each) or vehicle solution on rat hippocampal neurons cultivated for 25 days in vitro (div) on MEAs. (**d**-**g**) Diagrams illustrate either the acute influence (**d,e**) or the chronic influence (**f,g**) of montelukast, LTC4 and LTD4 on (i) the number of recorded spikes (NoSP), (ii) the percentages of spikes organized in bursts (%SpiB) and (iii) the degree of synchronicity indicated by the Cohen´s kappa value (kappa). Neither montelukast nor LTC4 and LTD4 had an acute or chronical influence on these parameters of neuronal function. Data are shown as mean ± SD (**a**) and mean ± s.e.m. (**d**-**g**). * $P < 0.05$; One-way ANOVA (**a,d-g**) with Bonferroni post hoc tests were performed. Scale bars in **a**: 50 µm. (**d**-**g**): One independent experiment with the following numbers of technical replicates was performed: montelukast-treated=6, vehicle-treated=8, LTC-4-treated=5, LTD-4-treated=5 and untreated= 5. *yg veh* young vehicle, *yg mtk* young montelukast, *old veh* old vehicle, *old mtk* old montelukast.

Supplementary Figure 4: Expression of GPR17 and CysLTR1 in different cell types of adult neurosphere-derived cells.

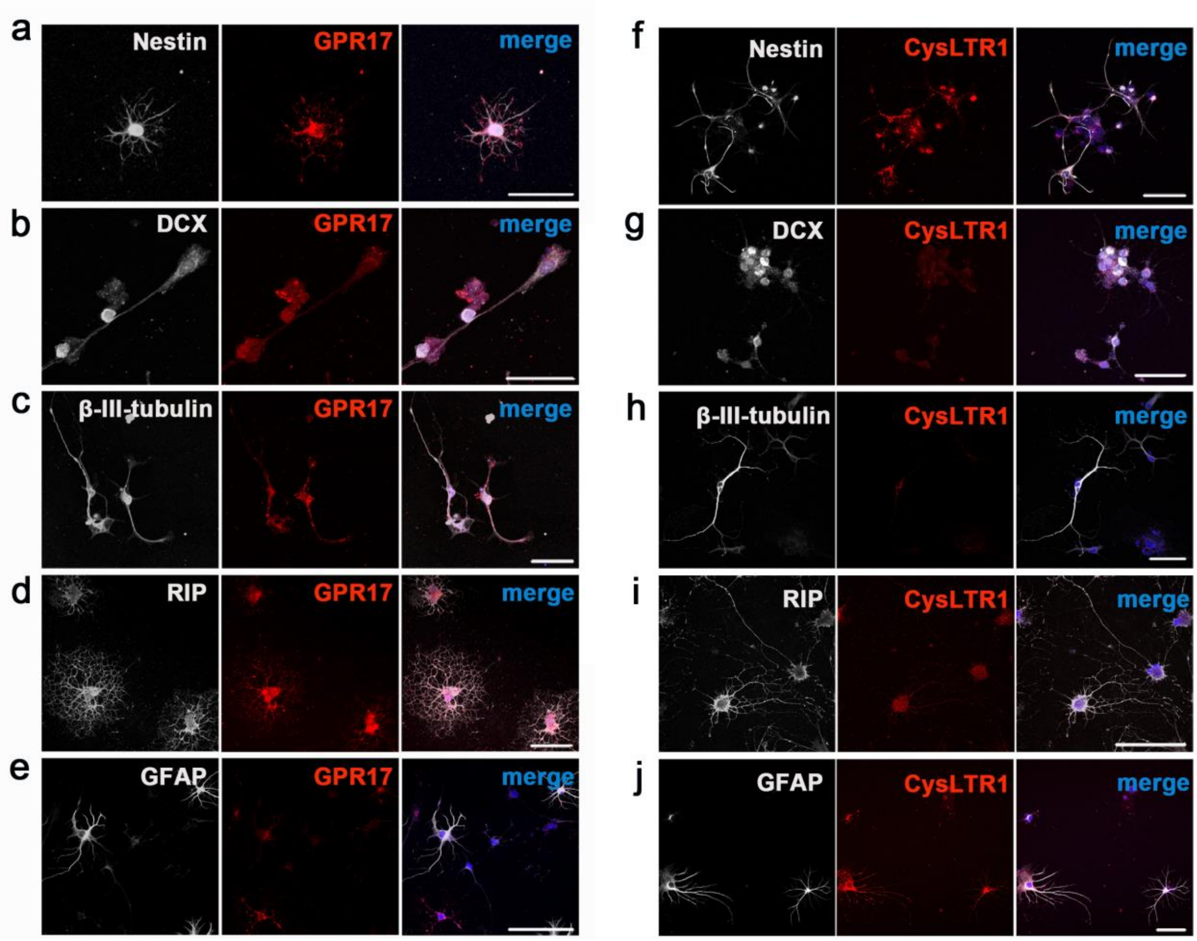

(**a**-**e**) GPR17 expression in adult rat hippocampal neurosphere derived cells *in vitro*. Nestin$^+$ cells (**a**), DCX$^+$ immature neurons (**b**) and beta-III-tubulin$^+$ young mature neurons (**c**) were moderately positive for GPR17. (**d**) Highest GPR17 staining intensity was assigned to RIP$^+$ oligodendroglial cells. (**e**) GPR17 expression was never observed in GFAP$^+$ astrocytes. **(f-j)** CysLTR1 expression profile *in vitro*. CysLTR1 immunoreactivity was localised to Nestin$^+$ neuronal stem cells (**f**), but was absent from DCX$^+$ immature neurons (**g**) and from beta-III-tubulin$^+$ young mature neurons (**h**). (**i**) Moderate CysLTR1 staining was observed in RIP$^+$ oligodendroglial cells, and strongest CysLTR1 staining intensity was observed in GFAP$^+$ astrocytes (**j**). Scales bars: 50 µm. Arrowheads indicate receptor co-localisation.

Supplementary Methods

Analysis of montelukast concentrations by mass spectrometry

4-month (n=6) and 20-month (n=4) old male wildtype F-344 rats received montelukast (10 mg kg^{-1}; dissolved in a 0.9% NaCl solution containing 10% ethanol) via oral gavage for seven consecutive days. One hour after the last gavage, blood was collected from every individual and serum was obtained by 1 hour incubation of the blood sample at 37°C and subsequent centrifugation at 13000 rpm. The supernatant was stored at -20°C until further processing. Immediately after blood collection, the animals were transcardially perfused with PBS to remove all blood from the body. Afterwards, cerebrospinal fluid (CSF) was collected from the cisterna magna, the brain was extracted, and all tissue was stored at -80°C.

Rodent brains were homogenised with an equal weight of water using a Bullet Blender (Next Advance). Aliquots of homogenate from each sample were transferred to a 96 well plate and 3 volumes of acetonitrile (Fisher) containing an internal standard (200 ng/mL tolbutamide, Sigma) were added to each. After centrifugation (15 min at 1500 g), supernatant (50 µL) was transferred to another 96 well plate and 100 µL of water added. Control brain tissue was treated in the same way to provide blank samples and calibration standards by spiking control brain homogenate with known amounts of montelukast. CSF was prepared for analysis by dilution with tolbutamide (200 ng mL^{-1}) in acetonitrile; artificial CSF was used as control matrix.

Human serum and CSF samples were collected and stored at -20°C for further analysis, which was approved by the ethic board of the Federal State Land Salzburg (415-E / 1160 / 4-2010).

Samples were analysed using an Agilent 1290 Infinity binary pump and autosampler with detection by an Agilent 6550 iFunnel QTof mass spectrometer (MS) (Agilent) using electrospray ionisation. The protonated molecules for montelukast and tolbutamide (m/z 586.2177 and m/z 271.1116) were extracted with ±15 ppm mass windows to generate chromatograms with suitable combinations of specificity and signal/noise. Sample aliquots (5

µL) were injected into a mobile phase initially of 98% water / 0.1% formic acid (Channel A) and 2% acetonitrile / 0.1% formic acid (Channel B) delivered at 0.4 mL /min to an Acquity BEH C18 50 x 2.1mm, 1.7 µm column. The column was maintained at 50°C in an Agilent Infinity column oven. The mobile phase composition was programmed to change linearly from 2% Channel B at 0.30 min post-injection up to 95% at 1.10 min, maintained at 95% until 1.75 min, and then returning to initial composition at 1.8 min. Column effluent was diverted to waste for the first 0.8 min post-injection to minimise source contamination.

Data processing was carried out using MassHunter software (v B.05.01, Agilent UK). Calibration curves were fitted using the simplest regression model to minimise back-calculated calibration standard concentration residuals over the range of study sample concentrations.

Immunohistochemistry

Free-floating sections were treated with 0.6% H_2O_2 in tris-buffered saline (TBS, 0.15M NaCl, 0.1M Tris-HCl, pH 7.5), rinsed in TBS and incubated in a blocking solution composed of TBS, 0.1% Triton X-100, 1% bovine serum albumin, and 0.2% teleostean gelatin (Sigma) for 1 hour. This buffer was also used during the incubation with primary antibodies, which were applied overnight at 4 °C. For chromogenic immunodetection, the sections were washed extensively and further incubated for 1 hour with a biotin-conjugated species-specific secondary antibody. Sections were then incubated for 1 hour in a peroxidase-avidin complex solution (Vectastain Elite ABC kit; Vector Laboratories). The peroxidase activity of immune complexes was revealed with a solution of TBS containing 0.25 mg/mL 3,3 diaminobenzidine (Vector Laboratories), 0.01% H_2O_2, and 0.04% $NiCl_2$. The following antibodies and final dilutions were used. Primary antibodies: rabbit anti-claudin-5 (1:300, ab53765, Abcam), rabbit anti-cFos (1:1000, Sc-52, Santa Cruz), rabbit anti-Synaptophysin (1:500,YE269, Abcam). Secondary antibodies: donkey anti-rabbit Alexa 488 (1:1000, Invitrogen), donkey-anti-rabbit biotinylated (1:500, Jackson Immuno Research). Chromogenic immunodetection

was photodocumented using a Zeiss Axioplan microscope (Zeiss,) equipped with the Zeiss AxioVision imaging system. Nuclear counterstaining was performed with 4', 6'-diamidino-2-phenylindole dihydrochloride hydrate at 0.25 µg/µl (DAPI; Sigma). Epifluorescence observation was performed on a confocal scanning laser microscope (LSM 710, Zeiss) with LSM software (ZEN 2011).

To analyse density of synaptophysin immunostaining in the cortex and hippocampus, the surface labelled with anti-synaptophysin was estimated. For both brain regions, 4 randomly selected visual fields per animal were photographed with a 20x objective and a resolution of 1024 × 1024 pixels using a confocal scanning laser microscope (LSM 710, Zeiss) with LSM software (ZEN 2011). Images were converted to grey scale (Adobe Photoshop CS2, Adobe, San Jose USA) prior to the selection of a detection threshold. The percentage of pixels containing labelling was quantified for every picture using ImageJ 1.45 s (ImageJ website: http://imagej.nih.gov/ij/).

Multielectrode array (MEA) recordings of hippocampal neuronal networks

Electrophysiological data were collected from cells plated on 6-well microelectrode array (MEA) dishes. Each well contains a square grid of 9 planar Ti/TiN microelectrodes 30 µm in diameter, spaced 200 µm apart (Multichannel Systems). Hippocampi dissected from embryonic day 18 (E18) Sprague Dawley rats were shipped to the Paracelsus University Salzburg from Brain Bits (Springfield, Illinois) in Hibernate B solution and were stored at 4°C until use. Directly after arrival (i.e. within 24 hours), the tissue was dissociated enzymatically in a solution of papain (20 units/ml; Worthington Biochemical) and deoxyribonuclease 1 (2000 units ml^{-1},) in GlutaMax-supplemented DMEM (Invitrogen) at 37 °C for 45 min before being triturated 10x gently and pelleted for 3 min at 1500 rpm. The pelleted cells were re-suspended in 'neuronal culture media' consisting of B27-Neurobasal medium supplemented with B27 (1%), GlutaMax (endc. 200 mM) and penicillin/streptomycin-mix (endc. 200 mM) (all from Invitrogen) and plated by placing a 15 µl droplet of neuronal culture media containing

15000 cells in the center of PDL-laminin-coated MEA dishes. After 2 hours, 300 µl of neuronal culture media were added to each well. Half media exchanges were performed twice a week.

After 3 weeks of neuronal maturation and neuronal network development, either montelukast (endc. 15 µM, diluted in H_2O), LTC4, LTD4 (100 nM, diluted in ethanol) or same volumes of vehicle solution (ethanol or H_2O) were added to the cultivation media. After 3 min of incubation, 3x 2 min consecutively recordings with pauses of 3 min as well as after 2 hours were performed to determine the acute impact on neuronal network activity. To determine the chronic impact on neuronal network activity, MEA measurements were performed 24 hours, 48 hours, 72 hours, 7 days, 10 days, 14 days, 21 days after initial application. Half media exchanges (150 µl media out / 150 µl media with either montelukast, LTD4, LTC4, vehicle) were performed twice a week.

Recordings were performed at 37°C (head stage heating). Extracellular potentials were recorded simultaneously on MEAs and all spike waveforms were stored separately and manually inspected for artefacts. The burst detection relied on an entropy-based algorithm. Interburst intervals were determined as time differences between the first spikes of subsequent bursts. Bursts were also checked by inspection for plausibility. We examined spike rate, burst rate, duration of bursts, interburst interval, interspike interval, and interspike interval inside a burst as well as the number of spikes per burst, percentages of spikes organized as bursts. Spontaneous spike rate was averaged over all electrodes and over the whole recording time (2 min for each measurement). The ratio of spikes organized in bursts describes the pattern of activity. Temporal synchronicity of spike activity across all electrodes was determined by binning all spikes into time intervals of 10 ms. For each pair of active electrodes, the spikes in non-overlapping bins were counted. A high percentage of bins without spikes on any electrode (empty bins) corresponds to weak activity or pronounced synchronicity across channels. For all pairs of active electrodes, the agreement coefficient Cohen's kappa was calculated, which measures the degree of coincidence of spikes on both

electrodes exceeding the chance-expected coincidence assuming uncorrelated spike activity. Theoretically, kappa may vary in the range -1 to +1.

Neuronal progenitor cell (NPC) differentiation

In order to analyse the expression of GPR17 and CysLTR1 in neuronal progenitor cells and in NPCs differentiated into neurons, astrocytes and oligodendroyctes, NPCs were plated on poly-ornithine (100 µg ml^{-1}) and laminin (5 µg ml^{-1}) -coated (Sigma) glass coverslips at a density of 25000 cells per cm^2 in NB-A medium for 24 hours. For immunocytochemical analysis of neuronal stem and progenitor cells and of differentiated neurons, NPCs were incubated in NB-A/B27 medium without any growth factors for 1, 4 and 7 days, respectively, then fixed for 30 min with 4% paraformaldehyde and processed for immunofluorescence. To provoke NPC differentiation into astrocytes and oligodendrocytes, NPCs were incubated in DMEM-knockout medium (Gibco BRL) with 20% of serum replacement (Invitrogen) plus 10% FBS (Lonza) (astrogenic stimuli) or DMEM knockout medium with 20% serum replacement without serum (oligodendrocyte differentiation) for 7 days. After the incubation period, cells were fixed for 30 min with 4% paraformaldehyde and processed for immunofluorescence.

Immunocytochemistry for neurosphere-derived cells

For immunocytochemical analysis of adult rat NPCs, fixed cells were washed in TBS (0.15 M NaCl, 0.1 M TrisHCl, pH 7.5), then blocked with a solution composed of TBS, 1% bovine serum albumin (BSA) and 0.2% Teleostean gelatin (Sigma) (fish gelatin buffer, FSGB). The same solution was used during the incubations with antibodies. Primary antibodies were applied overnight at 4°C. Fluorochrome-conjugated species-specific secondary antibodies were used for immunodetection. The following antibodies and final dilutions were used. Primary antibodies: mouse anti-beta 3 tubulin (1:500, G7121, Promega),

rabbit anti-CysLTR1 (1:500, SP4109P, Acris), guinea pig anti-doublecortin (1:2000, AB2253, Millipore), guinea pig anti-GFAP (1:1000, GP52, Progen), rabbit anti-GPR17 (1:400, 10136, Cayman), mouse anti-rat Nestin (1:500, 556309, Pharmingen), mouse anti-RIP (1:1000, NS-1, Millipore). Secondary antibodies: donkey anti-rabbit Alexa 568, donkey anti-goat, -guinea pig, -mouse Alexa 647 (all 1:1000, Invitrogen). Nuclear counterstaining was performed with 4', 6'-diamidino-2- phenylindole dihydrochloride hydrate at 0.25 µg/µl (DAPI; Sigma). Specimens were mounted on microscope slides using a Prolong Antifade kit (Molecular Probes). Epifluorescence observation was performed on a confocal scanning laser microscope (LSM 710, Zeiss,) with LSM software (ZEN 2011).
